# Foxp3 inhibitory peptide encapsulated in a novel CD25-targeted nanoliposome promotes efficient tumor regression in mice

**DOI:** 10.1038/s41401-024-01338-0

**Published:** 2024-07-29

**Authors:** Alejandro Serrano, Noelia Casares, Iñaki F. Trocóniz, Teresa Lozano, Juan J. Lasarte, Sara Zalba, María J. Garrido

**Affiliations:** 1 Department of Pharmaceutical Sciences, School of Pharmacy and Nutrition, University of Navarra, Pamplona, Spain; 2 Navarra Institute for Health Research (IdisNA), Pamplona, Spain; 3 Program of Immunology and Immunotherapy, CIMA, Pamplona, Spain

**Keywords:** Treg cells, CD25, lipid nanoparticle, PEG chain lengths, post-insertion, translation approach

## Abstract

P60, a Foxp3 inhibitory peptide, can hinder the regulatory T cell (Treg) activity and impair tumor proliferation. However, low systemic stability and poor specificity have led to daily dosing to achieve therapeutic effect. Therefore, this study aims to improve P60 stability and specific delivery through its encapsulation in liposomes targeting CD25, constitutively expressed in Tregs. P60 liposomes formulated with DSPE-PEG_750_ or DSPE-PEG_2000_ were incubated with DSPE-PEG_2000_-Maleimide micelles conjugated to Fab’ fragments of anti-CD25 to develop two targeted formulations or immunoliposomes (IL): IL-P60_2000_ (DSPE-PEG_2000_ only) and IL-P60_750_ (combining DSPE-PEG_750_ and DSPE-PEG_2000_). P60 encapsulation efficiency was 50%–60% irrespective of PEG chain length. Treg uptake was 2.5 and 14 times higher for IL-PEG_750_ compared with IL-PEG_2000_ and non-targeted liposomes, respectively, in in-vitro assays. In fact, IL-P60_750_ allowed CD8^+^  T cells ex-vivo proliferation in presence of Treg at doses 10–20 times lower than for free P60. Antitumor response of P60 and IL-P60_750_ in monotherapy and combined with anti-PD-1 was evaluated in MC38 and LLCOVA tumor bearing mice. In MC38 model, IL-P60_750_ monotherapy induced total tumor regression in 40% of mice reaching 100% for anti-PD-1 combination. This effect was associated with a significant increase in activated CD8^+^ T cells in tumors. Notably, IL-P60_750_ also inhibited human Treg in ex-vivo assay, showing the translational capability of this formulation. In conclusion, IL-P60_750_ formulated with different PEG chain lengths, has demonstrated antitumor efficacy by selective inhibition of Treg activity and enhances the effect of anti-PD1. Altogether, this novel IL represents a promising nanoplatform for cancer immunotherapies.

## Introduction

The presence of immunosuppressive cells in the tumor microenvironment (TME) is a mechanism that dampers the antitumor immune response induced by current immunotherapies [1]. Regulatory T cells (Tregs), a subset of CD4^+^ T cells, constitute one of these cellular immune suppressive mechanisms [2]. They promote the secretion of inhibitory cytokines (IL-10, TGF-β, and IL-35), capturing stimulatory cytokines such as IL-2 while inhibiting the maturation of antigen-presenting cells [3]. Consequently, Tregs can reduce the activity of CD8^+^, CD4^+^ T cells and Natural Killer (NK) cells, leading to tumor progression [4]. Indeed, high levels of Treg infiltration in the TME is associated with a poor prognosis and immunotherapy failure in cancer patients [5]. In this context, the inhibition of Tregs is essential for immunotherapy efficacy [6, 7].

The suppressive function of Tregs is controlled by the transcription factor Forkhead box P3 (Foxp3), responsible for Treg activity and proliferation [8]. Upon activation, Foxp3 dimerizes and translocates to the nucleus, interacting with hundreds of genes that modulate important cell processes, including its own transcription. In this regard, P60, a linear 15 amino acid peptide, has demonstrated selective binding capacity to Foxp3, downregulating its nuclear translocation, and thereby inhibiting Treg activity [9]. However, the low in-vivo stability of P60, with a very short plasma half-life and the poor selectivity by Treg cells, limit its clinical translation [10]. To overcome these, we proposed the encapsulation of P60 in a nanoliposome formulation to increase the systemic stability.

Liposomes have shown to improve pharmacokinetics and tumor accumulation via the enhanced permeability and retention (EPR) effect, increasing cargo release in the TME [11, 12]. Furthermore, the functionalization of liposomes by coupling different ligands on the surface, allows the recognition of specific targets in cells of interest, increasing the selective binding and delivery of their payload to the cell [13]. This strategy improves the intracellular bioavailability of the encapsulated agent, and thereby the therapeutic response [14]. Immunoliposomes (IL), a type of targeted liposomes, are prepared by conjugating ligands such as whole antibodies or antibody fragments to the surface of liposomes. In the present work, anti-CD25, in particular the monovalent variable fragment (Fab’-CD25), was chosen to formulate IL for Treg targeting [13, 15]. Treg cells constitutively express CD25^+^ (IL-2 receptor) generating high avidity for IL-2 [16].

**Fig. 1:**
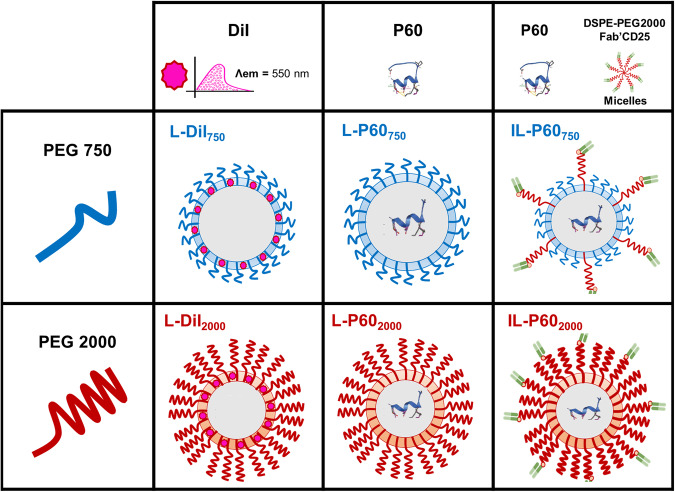
Encapsulated peptide formulations. Schematic representation of the liposomal formulations (non-targeted and targeted) developed in this work incorporating PEG with short and long chains.

Of note, ILs are seriously affected by the well-known “PEG dilemma”, a phenomenon in which PEG polymer present in the formulation hampers ligand recognition of targeted nanoparticles, reducing the efficiency of binding [17]. To address this challenge, we designed two types of targeted P60 liposomes utilizing the post-insertion method [18, 19]. This method provides the integration of targeted micelles formulated with DSPE-PEG_2000_ conjugated with the ligand, Fab’-CD25, into the membranes of P60 loaded liposomes previously prepared using PEG with different chain length (2000 or 750). The initial targeted P60 liposomes were formulated with conventional DSPE-PEG_2000_ combined with targeted micelles; whereas the second set of liposomes incorporated DSPE-PEG_750_ with targeted micelles. The latter formulation aims to enhance Treg targeting by reducing the PEG coating thickness through the use of shorter polymeric chains, PEG_750_, providing optimal flexibility to the ligands to interact with CD25^+^ and thus, enhancing the selective delivery of P60 to Treg cells (Fig. 1).

Therefore, the goal of this study is to develop P60 liposomes decorated with monovalent Fab’-CD25 fragments, to increase the in-vivo stability and selective delivery of P60 to Treg cells.

To our knowledge, this IL for Treg targeting represents a novel formulation to deal with one of the most relevant tumor immune escape mechanisms and may constitute an alternative strategy in the field of tumor immunotherapy.

## Materials and methods

### Materials

The lipids, hydrogenated soy L-α-phosphatidylcholine (HSPC), cholesterol (CH), 1,2-Distearoyl-sn-Glycero-3-Phosphoetanolamine-N-[Methoxy(Polyethylene Glycol) 2000] (Ammonium salt) (DSPE-PEG_2000_), 1,2-Distearoyl-sn-Glycero-3-Phosphoetanolamine-N-[Methoxy(Polyethylene Glycol) 750] (Ammonium salt) (DSPE-PEG_750_) and 1,2-Distearoyl-sn-Glycero-3-Phosphoethanolamine-N-[Maleimide(Polyethylene Glycol) 2000] (Ammonium salt) (DSPE-PEG_2000_-Mal) were purchased from Avanti Polar Lipids (Alabama, USA). PD-10 prepacked columns (Sephadex G-25M) were purchased from GE Healthcare (Chalfont, UK). Dialysis membrane (10 KDa MWCO), 1,1′-Dioctadecyl-3,3,3′,3′-tetramethyl-indocarbocyanine perchlorate (DiI), Hepes, Urea, ammonium molybdate, Tris, β-mercaptoethylamine (MEA), Amicon centrifugal filter units, ACK buffer, dialysis bag (12,000 MWCO), AB serum and sodium acetate were purchased from Sigma Aldrich (Madrid, Spain). Chloroform, D-glucose and perchloric acid (70%) were purchased from Scharlab (Barcelona, Spain). Ascorbic acid and methanol were purchased from Guinama (Valencia, Spain) and Merck (Darmstadt, Germany), respectively. Micro BCA Reactive Kit, immobilized pepsin, human interleukin-2 (hIL-2) and tris 2-carboxyethyl phosphine (TCEP) were purchased from Thermo Fisher Scientific (Massachusetts, USA). A-mouse-CD25 (Clone PC-61.53) and a-human-CD25 (Clone 7G7B6) were purchased from BioXcell (Lebanon, USA). Zombie NIR Fixable dye, a-CD45.2-Pecy5.5, a-CD25-PeCy7, a-NKp46-APC, PMA/Ionomycin, GolgiStop, GolgiPlug, a-IFNγ-PE, a-TNFα-PeCy7, a-CD45.2-APC, a-CD8-FITC, a-CD4-PeCy7, a-CD44-PeCy5.5, a-PD1-PB, a-NKp46-APC, a-F4/80-PB and a-Granzyme-PB were obtained from Biolegend (San Diego, CA). ELISPOT kit and Fixation/perm buffer were purchased from BD Biosciences (Heidelberg, Germany). RPMI-1640, Penicillin-streptomycin, PBS, Glutamine, 2-mercaptoethanol and FCS were provided by Gibco (Waltham, Massachusetts). Collagenase D and DNase-I were purchased from Roche (Basel, Switzerland). Dynabeads a-CD3/28 were provided by Dynal, Oslo, Norway. Finally, human regulatory staining Kit was obtained from eBioscience (California, USA) and X-vivo medium was purchased from Lonza (Basel, Switzerland).

### Development and characterization of pegylated P60 Liposomes

Peptide P60 2A5A (henceforth P60) with the sequence RAFQAFRKMWPFFAM was developed by the Center for Applied Medical Research (CIMA, Pamplona, Spain) [20].

Liposomes were prepared using the film-hydration method as described elsewhere [21, 22]. Briefly, HSPC:CH:DSPE-PEG_2000_ or HSPC:CH:DSPE-PEG_750_ lipids, at a molar ratio of 62:33:5, were disolved in chloroform:methanol (9:1 *v/v*) followed by rotary solvent evaporation at 40 °C (Büchi R-144, Switzerland) until film formation. P60 solution was prepared in Hepes buffer (10 mM Hepes, 150 mM NaCl, 5 mM EDTA; pH 6.7) or Urea buffer (10 mM Hepes, 5% *w/v* glucose, 7  M urea; pH 7.2) [10].

The film was hydrated with buffer solution for empty liposomes or with P60 solution at different peptide:lipid ratios (1:3, 1:4, 1:6, and 1:8 µmol/µg) for 20 min [20]. Liposomes were extruded through a series of polycarbonate membranes with one drain disk, starting with 200 nm and then changing to 100, 80, and 50 nm, respectively. To remove the non-encapsulated P60, liposomes solution was eluted using PD-10 columns (Sephadex G-25M, GE Healthcare, Madrid, Spain) and washed with Hepes buffer to purify P60 liposomes, L-P60_750_ or conventional L-P60_2000_. Empty fluorescent liposomes were formulated with 0.3% *w/w* of DiI following the same procedure (L-DiI_750_ and L-DiI_2000_) [23]. Formulations were stored at 4 °C until use.

The post-insertion method was used to formulate targeted liposomes [18, 19]. For that, murine or human anti-CD25 monoclonal antibody was enzymatically digested and processed to obtain the monovalent variable fragment of the antibody, Fab’-CD25 [23]. These fragments were incubated overnight at 4 °C under constant stirring with micelles of DSPE-PEG_2000_-Mal prepared in Hepes buffer to obtain Fab’-CD25 targeted micelles [23]. Finally, L-P60_750_ and L-P60_2000_ liposomes previously formulated were incubated with targeted micelles for 1 h at 55 °C at a 1:99 molar ratio (micelles/liposomes) to obtain the corresponding immunoliposomes (IL-P60_750_ and IL-P60_2000_) [18, 19]. Note that IL-P60_750_ included two different PEG chain lengths, 750 for stability and 2000 for ligand conjugation (Fig. 1).

Liposomes were characterized in terms of particle size, polydispersity index (PDI) and Zeta Potential by laser diffractometry using a ZetaSizer Nano Series system (Malvern Instruments, UK). Exclusion criteria were a size > 200 nm and/or PDI > 0.3. Lipid concentration was quantified by the Bartlett phosphate assay [24] using Agilent 8453 spectrophotometer (Agilent, Santa Clara, USA). The encapsulation of P60 was measured by the micro-BCA assay (Thermo Fisher, Madrid, Spain) [25] using a Powerwave XS Spectrophotometer (Biotek Instruments, Winooski, USA).

To that aim, free peptide diluted in Hepes buffer or Urea buffer was used to build standard curves. Linearity with urea buffer ranged from 0.97 μg/mL to 500 μg/mL establishing the limit of quantification (LOQ) of 0.97 μg/mL; linearity with Hepes buffer ranged from 1.95 μg/mL to 1000 μg/mL with a LOQ of 1.95 μg/mL.

Encapsulation efficiency (EE) was calculated according to Eq. 1, whereas the loading efficiency (LE) was calculated using Eq. (2):

1 $${EE}\left( \% \right)=\frac{{Fa}}{{Ia}}* 100$$

2 $${LE}\left({{{{\rm{\mu }}}}}g/{mL}\right)={Fa}-{Fb}$$

Where *Fa* represents the final amount of encapsulated peptide divided by the initial amount of peptide (*Ia*) and *Fb* represents the blank signal of empty liposomes, corrected by lipid concentration. The coupling efficiency of a-CD25-Fab’ was measured using a micro-BCA assay, and the number of ligands per liposome was calculated using the Avogadro number [23].

Images characterizing non-targeted and targeted liposomes morphology were performed using cryo-transmission Electron Microscopy (cryo-TEM) as detailed in Supplementary material (Fig. S1).

### Liposome stability and P60 release assay

Long-term stability of P60 liposomes kept at 4 °C was characterized in terms of particle size, PDI, and P60 EE. For that, at selected time points, aliquots of the different formulations were filtered through PD-10 columns to remove free P60, and samples were analyzed and quantified as mentioned above.

The release curve of P60 was assayed by dialysis [26]. Briefly, 1 mL of IL-P60_2000_ or IL-P60_750_ liposomes was placed in a semi-permeable dialysis bag with 10% FBS and immersed in 20 mL of PBS (1:20 *v/v*), at 37 °C with constant stirring (200 rpm). At different time points from 0 to 24 h, samples from the external medium were collected replacing the same volume to ensure sink conditions. Samples were measured by micro-BCA, and the accumulative release curve was built using the following equation (Eq. 3):

3 $${Release}( \% )=\frac{{Qa}}{{Qt}}\times 100$$

Where *Qa* represents peptide amount in each sample, and *Qt*, the total amount of P60 encapsulated in liposomes. The release rate of free P60 was used as a positive control, and the possible interaction of P60 with liposomes was tested by evaluating the release of free P60 in presence of empty liposomes.

### Cell lines and cell cultures

MC38 (murine colon cancer cell line) and LLCOVA (murine lung cancer cell line) were kindly provided by Dr. Melero and Dr. Ajona (CIMA, Pamplona, Spain). Both tumor cell lines were cultured in complete RPMI-1640 medium containing 10% FBS, penicillin-streptomycin, 2 mM glutamine, and 50 μM of 2-mercaptoethanol. Cell lines were regularly tested for mycoplasma (Lonza, Basilea, Switzerland). The choice of these two cell lines was guided by the varying levels of tumor-infiltrating Tregs found in the tumors generated following their in vivo injection in mice. The MC38 model exhibited a higher Treg infiltration [27]. Similar data have been reported by other authors [28, 29].

### Animals

C57BL/6 (6-week-old) female mice (B6-Foxp3EGFP/B6.Cg-Foxp3tm2 (EGFP) Tch/J) purchased from Jackson Laboratory (Maine, USA) were used in ex-vivo experiments. These Foxp3-GFP reporter mice co-express EGFP and the regulatory T cell-specific transcription factor Foxp3.

In vivo antitumor efficacy assays were performed using 6-week-old C57B6/J female mice supplied by Harlan (Barcelona, Spain).

Mice were housed in sterile plastic cages with enrichment elements and wood chips as bedding material. A maximum of six animals were assigned to each cage and maintained under standard conditions (25 °C, 50% relative humidity, 12 h dark/light) with sterile water and food *ad libitum*, at the animal facility of the Center of Applied Medical Research (CIMA, Pamplona, Spain).

All experiments were performed according to European animal care regulation, ARRIVE guidelines and the protocol approved by the Ethic Committee of the University of Navarra and Government of Navarra (Ref. 023-17).

### Ex-vivo liposome-Treg interaction

Treg cells were collected from Foxp3-GFP reporter mice spleens. To stimulate lymphocyte proliferation and the upregulation of CD25 expression, cells were cultured in presence and absence of a-CD3/28 Dyna beads at 1:2 bead*/*lymphocyte ratio during 24 h. Afterwards, spleen cells were incubated with 100 μM of non-targeted (L-DiI_750_ or L-DiI_2000_) or targeted liposomes (IL-DiI_750_ or IL-DiI_2000_) for 30 min or 4 h at 37 or 4 °C. The liposomal dose was optimized after testing different concentrations (data not shown). To evaluate the specific binding of targeted liposomes to Tregs, the same assay was performed in parallel in presence of free a-CD25 (5 μg/mL) able to block the receptor. Data acquisition was performed using a FACS Canto II flow cytometer (Becton Dickinson, New Jersey, USA) and analyzed using FlowJo software V10 (TreeStar Inc, Ashland, USA).

### Biodistribution of immunoliposome in tumor bearing mice

Foxp3-GFP reporter mice were subcutaneously inoculated with 5 × 10^5^ MC38 cells/100 μL PBS or 2 × 10^6^ LLCOVA cells/100 μL PBS (*n* = 12; 6 mice per cell line). Tumor volume was measured twice a week using an electronic caliper and calculated according to Eq. (4):

4 $${Tumor\; Volume}\left({{mm}}^{3}\right)=\frac{{Width}\times {{Length}}^{2}}{2}$$

When tumors reached a diameter of 8 mm, mice were randomly divided to receive empty fluorescent formulations (2 nmol of lipids), L-DiI_750_ or IL-DiI_750_, as a single intravenous dose. Blood samples, spleen, and tumors were collected 6 h after treatment. Lipid concentration and final time point of 6 h were chosen based on previous data from our group [23, 30].

Spleen and blood were homogenized in PBS while excised tumors were digested with 400 U/mL of Collagenase D and 50 μg/mL DNAse-I for 30 min at 37 °C, followed by incubation with ACK buffer to remove red cells. Cells from all organs were stained with Zombie NIR, a-CD45.2-Pe-Cy5.5, a-CD25-PeCy7 and a-NKp46-APC for 20 min at room temperature (RT). Samples were washed and resuspended in FACS buffer (5% FCS and 0.5% EDTA in PBS) for analysis. Data acquisition was performed using a FACS Canto II flow cytometer and analyzed using FlowJo. Antibodies and reagents used for cell staining were purchased from BD Biosciences (Heidelberg, Germany) and BioLegend (San Diego, CA, USA). A detailed list of antibodies and reagents is in Supplementary Table S1.

### Antitumor response induced by immunoliposomes in monotherapy

A total of 48 C57B6/J female mice (*n* = 8 mice/group) were subcutaneously inoculated with 5 × 10^5^ MC38 cells in 100 μL of PBS. Three days later, mice were randomly divided into six groups: (1) control or non-treated; (2) free P60 at 5 mg·kg^−1^·d^−1^ for 10 consecutive doses (i.p.); (3) free P60, 0.25 mg/kg every two days for four doses (i.p.); (4) L-P60_750_, 0.25 mg/kg of peptide every two days for four doses (i.v.); (5) IL-P60_750_, 0.25 mg/kg peptide every two days for four doses (i.v.); and (6) empty IL_750_ (lipid amount and posology as group 5).

Treatment efficacy was evaluated by measuring tumor growth over time and survival. The general welfare of animals was checked regularly to ensure normal behavior and the absence of injuries. Mice were sacrificed when tumors reached diameters ≥ 15 mm.

### Immunological profile induced by immunoliposomes in MC38 tumor bearing mice

A total of 48 C57B6/J female mice were subcutaneously inoculated with 5 × 10^5^ MC38 cells in 100 μl of PBS. Three days later, mice were randomly divided into six groups (*n* = 8 mice/group), according to the previous experimental design.

At day 15 after tumor cell inoculation, mice were sacrificed and spleen, tumor, and lymph nodes were collected and processed to evaluate T-cell response. This time point was chosen based on previous experimental assays (data not shown).

For surface markers, samples stained for 20 min at RT with Zombie NIR, a-CD45.2, a-CD8, a-CD4, a-CD44, a-NKp46, a-F4/80 and a-PD were analyzed by flow cytometry. For intracellular staining, cells were stimulated for 5 h with 50 ng/ml PMA and 1 μg/ml Ionomycin followed by GolgiStop and GolgiPlug. Then, cells incubated with Zombie NIR, fixed and permeabilized with the Fixation/perm buffer, were stained with a-IFNγ and a-Granzyme B antibodies for 20 min at RT before the analysis. For immune activation, IFNγ levels produced by T cells from the spleen (8 × 10^5^/well) were quantified by ELISPOT. In brief, the day before the experiment, a 96-well Elispot microtiter plate was coated with capture antibody overnight (1:200 dilution in PBS) at 4 °C. 24 h later, the plate was washed and blocked for 2 h by adding complete RPMI culture medium. T cells were stimulated for 24 h by incubation with MC38 cells, previously irradiated, at a 10:1 ratio (*lymphocytes/tumor*) at 37 °C. One day later, cells were washed and incubated for 2 h with biotinylated antibody (1:250) prepared in PBS and 10% FBS, followed by an incubation with streptavidin-HRP (1:100) for 1 h. Then, the substrate solution was added, and the reaction was stopped by adding water. Spots were counted using an automated ELISPOT reader (CTL, Aalen, Germany).

### Antitumor response induced by combinational immunotherapies

A total of 96 C57B6/J female mice (*n* = 48 mice/cell line) were subcutaneously inoculated with 5 × 10^5^ MC38 cells or 2 × 10^6^ LLCOVA cells in 100 μL of PBS. Three days later, mice randomly divided into six groups (*n* = 8 mice/group) received: (1) control or non-treated; (2) 5 mg·kg^−1^·d^−1^ of free P60 for 10 consecutive doses (i.p.); (3) 5 mg/kg of a-PD-1 every three days for three doses (i.v.); (4) IL-P60_750_ at 0.25 mg/kg of peptide every two days for four doses (i.v.); and (5) the combination of a-PD-1 and IL-P60_750_ as described in group 3 and 4.

Treatment efficacy was evaluated by measuring tumor growth over time and survival. The general welfare of animals was checked regularly to ensure normal behavior and the absence of injuries. Mice were sacrificed when tumors reached diameters ≥ 15 mm.

### Functional assays using human Treg cells

For this assay, Tregs were isolated from the umbilical cords of human donors.

Briefly, blood samples were sorted using a FACS Aria cytometer (BD Biosciences) to purify Tregs (CD4^+^/CD25^+^/CD127^-^). These cells were stimulated with a-CD3/CD28 beads at 1:4 bead/cell ratio in X-vivo medium supplemented with 10% AB human serum and cultured for 14 d under standard conditions. Typically, medium exchanges occurred every other day with fresh AB serum with 300 UI/ml hIL-2, keeping a concentration of 0.3 × 10^6^ cells/ml throughout the culture. On day 9, cells were re-stimulated with a-CD3/CD28 beads at a 1:1 ratio. On day 14, cells were analyzed by flow cytometry selecting those Tregs that expressed Foxp3 at approximately 70%. Experiments with human samples were approved by the Institutional Ethics Committee (ref. 2022/093).

For this experiment, IL-P60_750_ were formulated with human a-CD25-Fab’. Cells were incubated for 4 h at 37 °C with: (1) non-targeted 2.5 μM P60 liposomes (L-P60_750_); (2) targeted 2.5 μM P60 liposomes (IL-P60_750_) and (3) 25 μM free P60. Dose selection was based on previous in house data [20].

Then, Tregs were washed and co-cultured for 2 d at 37 °C with PBMCs, also obtained from human donors, in a 1:2 Treg/Teff ratio. The co-culture was stimulated with a-CD3/CD28 at a 1:10 bead/Teff ratio. At the end of experiment, on day 2, cell proliferation was measured by the incorporation of ^3^H-thymidine. Briefly, 0.5 μCi of [methyl-^3^H] thymidine was added to each well and incubated overnight. Cells were harvested by Filtermate-96 harvester (Packard Instrument, Meriden, CT, USA) and the incorporated radioactivity was measured using a scintillation counter TopCount (Packard Instrument) as a readout of T-cell proliferation [31].

### Statistics

Data are expressed as means with standard deviation (SD) or standard error of the mean (SEM). Normal distribution of data was determined by Shapiro-Wilk test. Statistical analysis was performed using a one-way Kruskal-Wallis test to compare all groups together, followed by the Wilcoxon-Mann-Whitney test to check for differences between groups (two by two). For survival curves, Mantel-Cox statistical analysis was performed. All analyses were performed using GraphPad Prism, version 8.0.2. The statistical significance level was set at *P* < 0.05.

## Results

### An optimization of liposomes encapsulating P60 was achieved using the urea chaotropic method

Table 1 summarizes the physicochemical characteristics of non-targeted liposomes formulated using the different conditions.

**Table 1:** Summary of P60 liposomes characteristics, particle size, polydispersity index (PDI), encapsulation efficiency (EE), and loading efficiency (LE) according to different scenarios: hydration buffers; lipid: peptide ratios.

	L-P60_2000_				L-P60_750_			
	Size (nm)	PDI	EE (%)	LE (µg/mL)	Size (nm)	PDI	EE (%)	LE (µg/mL)
Buffer								
Hepes	114.2 ± 0.721	0.033 ± 0.010	18.76 ± 0.35	16.05 ± 0.35	124.5 ± 2.06	0.048 ± 0.021	23.24 ± 4.60	23.40 ± 5.61
Urea 3.5 M	112.47 ± 3.40	0.048 ± 0.017	62.63 ± 2.57^a^	85.87 ± 10.15	115.23 ± 2.45	0.047 ± 0.013	58.81 ± 3.59^b^	75.07 ± 7.09
Urea 7 M	113.90 ± 4.88	0.040 ± 0.026	58.63 ± 1.93	89.85 ± 9.55	118.74 ± 2.65	0.05 ± 0.026	53.70 ± 6.98	64.14 ± 4.53
Lipid:Peptide (µmol:µg)								
1:3	116.80 ± 2.83	0.020 ± 0.004	43.22 ± 0.52	29.56 ± 0.58	98.38 ± 4.8	0.063 ± 0.026	34.01 ± 2.46	29.00 ± 0.62
1:4	118.3 ± 2.97	0.032 ± 0.007	46.42 ± 4.22	45.31 ± 2.14	118.37 ± 4.31	0.029 ± 0.010	44.60 ± 6.3	46.59 ± 5.38
1:6	112.47 ± 3.40	0.048 ± 0.017	61.03 ± 3.01^c^	85.87 ± 10.15	115.23 ± 2.45	0.047 ± 0.013	56.26 ± 5.70	80.13 ± 8.62
1:8	117.0 ± 4.53	0.068 ± 0.006	45.27 ± 1.37	94.44 ± 2.87	118.37 ± 4.31	0.063 ± 0.026	53.16 ± 1.78	80.62 ± 2.70
Stability (day)								
0	113.18 ± 3.84	0.042 ± 0.021	58.48 ± 1.36	91.51 ± 3.89	116.99 ± 2.99	0.049 ± 0.027	49.75 ± 1.92	80.13 ± 8.62
15	116.0 ± 1.73	0.041 ± 0.002	55.74 ± 0.53	39.32 ± 6.29	114.7 ± 0.80	0.088 ± 0.051	42.07 ± 2.91	42.54 ± 2.53
30	112.28 ± 2.65	0.067 ± 0.015	51.86 ± 4.42	22.71 ± 6.09	118.08 ± 4.0	0.082 ± 0.044	36.60 ± 1.48	34.36 ± 2.37
60	108.8 ± 0.902	0.046 ± 0.015	50.69 ± 3.32	21.98 ± 5.05	113.6 ± 1.75	0.049 ± 0.025	36.20 ± 1.70^d^	22.36 ± 4.76

The long-term stability for liposomes formulated with 3.5 M urea buffer and 1:6 (lipid:P60) ratio was also assayed at 4 °C for 60 d. Data represent the average ± standard deviation of three independent batches.

^a^L-P602000 Hepes vs. 3.5 M (*P* < 0.05).

^b^L-P60750 Hepes vs. 3.5 M (*P* < 0.05).

^c^L-P602000 1:6 μmol:μg ratio vs. the rest of ratios (*P* value < 0.01).

^d^L-P60750 Day 0 vs. Day 60 (*P* value < 0.05).

The EE of P60 was significantly increased for the urea buffer ~60% (*P* < 0.05) compared to Hepes buffer ~20%. For urea buffer, no statistical differences (*P* = 0.342) in the liposomal characteristics were found between 7 and 3.5 M concentrations, selecting the lowest concentration to formulate liposomes. For the lipid/peptide ratio, 1:6 μmolːμg resulted the optimal to provide the highest EE (61.03%; *P* < 0.01). Therefore, this ratio was also selected to prepare all the formulations used in this study. In addition, PEG length (2000 or 750) posed no statistically (*P* = 0.217) differences on characteristics of liposomes in terms of particle size, PDI and EE, supporting the robustness of the methodology to prepare liposomes.

Furthermore, the long-term stability of liposomes kept at 4 °C was evaluated and compared to fresh formulations, showing no differences in EE, particle size or PDI over 60 d. Although P60 accumulative leakage during this time was slightly higher (27%) for L-P60_750_ than for L-P60_2000_ (14%), this difference did not reach statistical significance (*P* = 0.33) (Table 2).

**Table 2:** Cumulative release curve of P60, free and encapsulated in liposomes formulated using DSPE-PEG2000 or DSPE-PEG750.

Accumulative Release (%)					
Formulation	5 min	15 min	2 h	4 h	24 h
Free P60	31.63 ± 1.55	38.02 ± 6.26	73.04 ± 3.65	84.84 ± 6.78	91.84 ± 3.13
L-P60_2000_	―	0.48 ± 0.67	2.30 ± 3.25	4.80 ± 2.86	11.59 ± 0.75
L-P60_750_	2.89 ± 2.74	3.18 ± 1.67^a^	11.14 ± 1.11	12.41 ± 0.80	20.57 ± 1.06^b^

This assay was carried out in presence of FBS (10%) at pH 7.4 and 37 °C. Data correspond to the mean ± SD of three independent batches (*n* = 3).

^a^DSPE-PEG_2000_ vs. DSPE-PEG_750_ at 15 min (*P* = 0.23).

^b^DSPE-PEG_2000_ vs. DSPE-PEG_750_ cumulative release at 24 h (*P* < 0.05).

ILs formulated by post-insertion method showed similar physicochemical characteristics to non-targeted formulations (Table 3), demonstrating no effect of the ligand attachment (Fab’-CD25). In addition, this result was also observed for ILs formulated with both, human and murine Fab’-CD25, demonstrating the robustness of this method ( ~ 100 nm and PD1 < 0.1 in both cases).

**Table 3:** Characterization of final selected liposomal formulations.

Formulation	Size (nm)	PDI	LE (µg/mL)	EE (%)
L-P60_2000_	112.4 ± 3.4	0.048 ± 0.017	85.8 ± 10.1	62.6 ± 2.6
L-P60_750_	115.2 ± 2.4	0.047 ± 0.013	80.1 ± 8.6	58.8 ± 3.6
IL-P60_2000_	82.8 ± 0.7	0.063 ± 0.006	92.6 ± 4.7	49.9 ± 2.5
IL-P60_750_	97.9 ± 9.5	0.073 ± 0.045	71.1 ± 9.3	53.8 ± 1.6
Human IL-P60_750_	108.9 ± 1.3	0.121 ± 0.018	73.2 ± 5.6	59.7 ± 3.2

Data represent the mean ± SD of three independent batches (*n* = 3).

*LE* loading efficiency, *EE* encapsulation efficiency; *L-P60*_2000_ P60 loaded non-targeted liposomes formulated with DSPE-PEG_2000_, *L-P60*_750_ P60 loaded non-targeted liposomes formulated with DSPE-PEG_750_, *IL-P60*_2000_ immunoliposome encapsulating P60 and formulated with DSPE-PEG_2000_, *IL-P60*_750_ immunoliposome encapsulating P60 and formulated with DSPE-PEG_750_.

The efficiency of ligand conjugation was calculated as 42%, representing 51.7 ± 9.02 Fab’-CD25 molecules per liposome (assuming a Fab’ molecular weight of 40 kDa). Since conjugation was associated in all cases with PEG_2000_-Maleimide micelles, this process was independent of the polymer chain length of P60 preformed liposomes.

Cryo-TEM images showed an unilamellar spherical morphology for both, LP-60_750_ and IL-60_750_, formulations. In addition, particle size determined by laser diffractometry was according to these images (supplementary material, Fig. S1).

### Ex-vivo data showed higher Treg interaction and internalization of ILs formulated with PEG_750_

Foxp3-GFP^+^ Treg cells isolated from the spleens of Foxp3-GFP^+^ reporter mice were exposed to targeted (IL-DiI) and non-targeted (L-DiI) liposomes at 4 and 37 °C to discriminate between passive cell interaction and active cell internalization, respectively (gating strategy is shown Fig. S2).

**Fig. 2:**
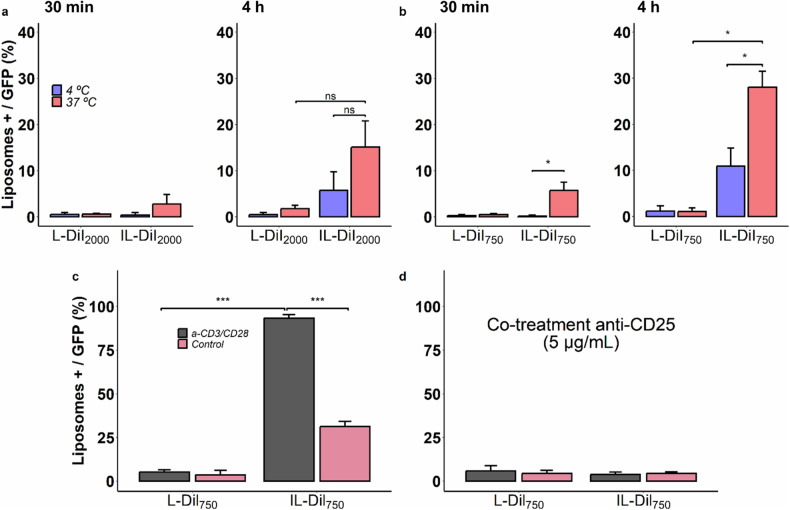
Foxp3^-^GFP^+^ Treg cells exposed to different fluorescent empty targeted and non-targeted formulations. **a** Liposomes formulated with DSPE-PEG_2000_ (IL-DiI_2000_) at 4 and 37 °C for 30 min and 4 h; (**b**) liposomes formulated with DSPE-PEG_750_ (IL-DiI_750_) at 4 and 37 °C for 30 min and 4 h; (**c**) interaction of IL-DiI_750_ with control cells or without stimulation (red bars) or overexpressing CD25 after stimulation (using a-CD3/CD28 antibodies) (gray bars); (**d**) selective interaction of IL-DiI_750_ to CD25 assayed in presence of a-CD25 to block IL-2 receptor. Bars represent the mean ± SD of three independent experiments (*P < 0.05; ***P < 0.001).

For ILs passive and active interactions were time-dependent regardless of PEG chain length (2000 or 750), (Fig. 2a, b). However, non-targeted liposomes were not affected by any experimental conditions, exposure time or temperature.

In the case of targeted liposomes, after 4 h of exposure, differences between cell interaction (4 °C) and active uptake (37 °C) reached statistical significance for IL-DiI_750_, (Fig. 2b; *P* = 0.045). Furthermore, fluorescent liposomal signal in Tregs was two times higher for IL-DiI_750_ than for IL-DiI_2000_, suggesting a better availability of ligands to recognize receptors when ILs combine two PEG lengths.

To demonstrate selectivity and specificity for CD25 expressed on Treg membrane, Foxp3-GFP^+^ Treg cells were split into non-stimulated (control) or a-CD3/28 stimulated groups and exposed to: (i) fluorescent liposomes or (ii) fluorescent liposomes together with the free anti-CD25. Non-targeted formulation interaction was not affected by the upregulation of CD25 upon cell stimulation. In contrast, the uptake of IL-DiI_750_ reached 30% in control cells and 100% in stimulated, demonstrating a dependence on receptor expression (Fig. 2c).

In line with these results, the co-treatment with free a-CD25, able to bind and block IL-2 receptor, totally inhibited the IL-DiI_750_ uptake by Treg cells, demonstrating the selectivity of this targeted liposome for the CD25 molecule (Fig. 2d).

### IL-DiI_750_ was selectively captured by Treg and NK cells

**Fig. 3:**
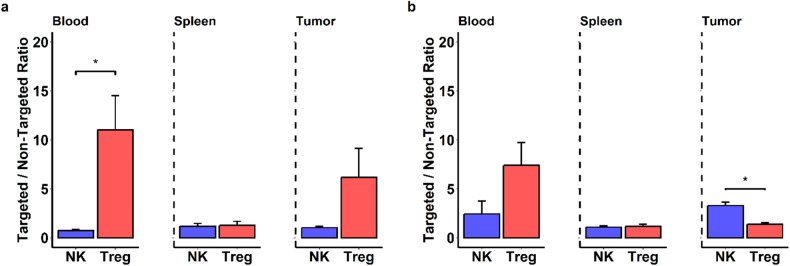
Biodistribution of fluorescent liposomes after i.v. administration to mice. **a** Relative fluorescent signal in Treg and NK cells in different tissues of mice inoculated with MC38 (*n*=6) and (**b**) relative fluorescent signal in Treg and NK cells in different tissues of mice inoculated with LLCOVA tumor cells (*n* = 6). Samples collected from blood, spleen and tumor tissue were analyzed by flow cytometry. Data were expressed as the relative uptake of targeted liposomes vs. non-targeted (IL-Dil_750_ Uptake) / (L-Dil_750_ Uptake) for comparison of the different levels found in the tissues. Bars represent the mean ± SD of three independent samples (*P < 0.05).

In-vivo biodistribution of targeted and non-targeted fluorescent liposomes was evaluated 6 h after intravenous administration in mice bearing MC38 and LLCOVA tumors. ILs were preferentially captured by CD25 expressing Treg and NK cells in blood and tumor for both models in comparison with non-targeted liposomes (Fig. 3). This different behavior between both formulations was also observed in MC38 tumors analyzed by in vivo imaging system (IVIS) (supplementary material Fig. S3).

In addition, the uptake of ILs by circulating Treg was 7–10 times higher compared to non-targeted formulations in both models. However, in Treg infiltrating MC38 tumors, the uptake of IL was 6 times higher than for non-targeted, while in LLCOVA tumors ILs were mainly captured by NK cells, reaching a fluorescent signal 3 times higher than for non-targeted (Fig. 3b and Fig. S4).

### IL-P60_750_ exerted a better antitumor immune response than free P60

**Fig. 4:**
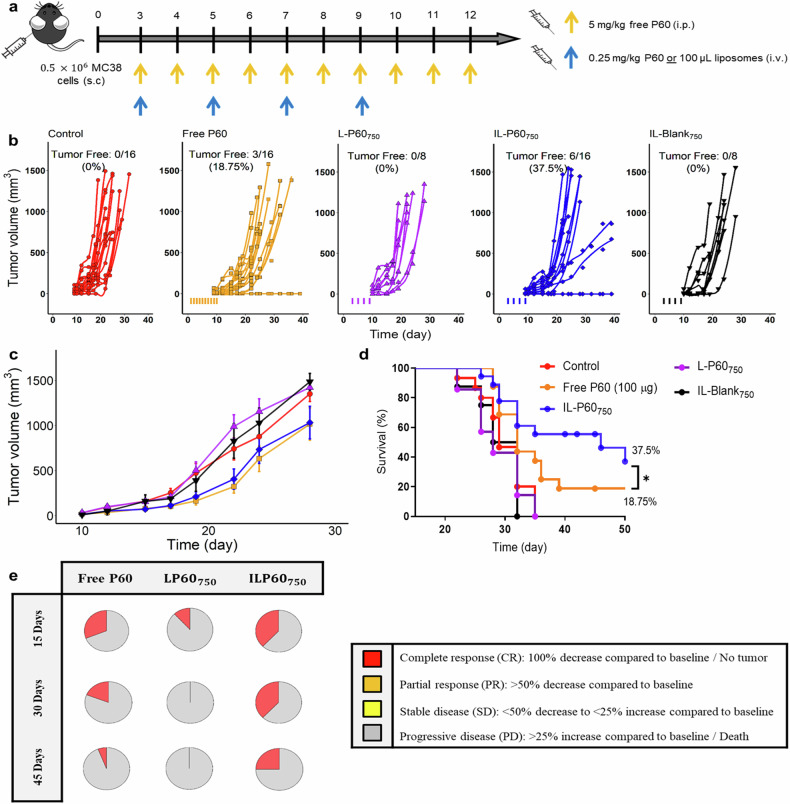
Antitumor in-vivo response assayed in MC38 tumor-bearing mice. **a** Experimental design scheme; (**b**) individual tumor growth kinetics induced by the different treatments; (**c**) time profiles of tumor growth computed as the mean ± SEM for each treatment; (**d**) long-rank survival curves; (**e**) evolution of tumor size based on clinical RECIST criteria [48]. Data corresponding to control, free P60 and IL-P60 groups comprised 16 mice/group (from two different experiments to increase the accuracy of findings); whereas L-P60 and empty ILs comprised 8 mice/group. (*P value < 0.05).

The antitumor response of IL-P60_750_ was first evaluated in MC38 murine model. In this model, mice receiving non-targeted P60 and empty CD25 targeted liposomes showed similar tumor progression to non-treated animals, demonstrating no therapeutic effect. However, the administration of free P60 (total dose of 1 mg/mouse) and IL-P60_750_ (total dose of 20 μg/mouse) induced total tumor regression in approximately 20% and 40% of animals, respectively (Fig. 4b). This difference resulted statistical significance and had an impact on the survival of animals (Fig. 4d, *P* = 0.039). In fact, when applying the RECIST criteria, P60 activity was clearly different depending on the formulation assayed (Fig. 4e). Hence, although treatments with P60 showed an initial response, only free peptide and ILs were able to maintain the effect for 30 days after finishing the treatment.

Notable, no animals showed signs of toxicity such as rapid loss of body weight or the presence of ulcers.

**Fig. 5:**
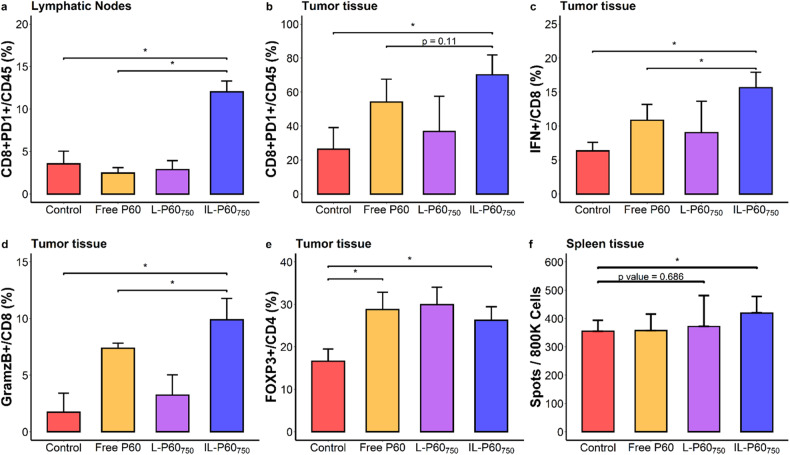
Immune response measured in MC38 tumor-bearing mice (*n* = 4 mice/group) at day 15 after starting the different treatments. **a** Activated effector CD8^+^ T cells (CD8^+^PD1^+^) in lymphatic nodes; (**b**) in tumor tissue; (**c**) IFNγ levels in tumor; (**d**) GramzB^+^ expression in tumor CD8^+^ and (**e**) FOXP3^+^ expression in CD4^+^ T cells in tumor; (**f**) IFNγ levels in spleen measured by ELISPOT assay. Bars represent mean ± SD (*P value < 0.05).

The immunological profiles were evaluated in MC38 tumor-bearing mice at day 15 after dose administration. Activated effector CD8^+^ T cells were significantly increased in lymph nodes (*P* = 0.049) and tumors (*P* = 0.029) in mice treated with IL-P60_750_ in comparison with mice receiving other treatments (Fig. 5a, b). Both, ILs and free P60 (at a dose 50 times higher than encapsulated) were able to increase statistically IFNγ levels and Granzyme B expression in tumor CD8^+^ T cells (Fig. 5c, d). This immunostimulatory effect was also observed systemically, as evidenced by the increase in tumor-specific IFNg-producing cells in the spleens of IL-P60_750_ mice treated. (Fig. 5f).

Finally, all treatments led to a slight increase in the number of Foxp3^+^CD4^+^ T cells in tumor, a fact that highlights the inhibitory effect of P60 on Foxp3 activity rather the depleting cells that is associated with the development of autoimmunity (Fig. 5e). Hence, our data support the improved immunostimulatory capacity of P60 when encapsulated in ILs, which in combination with other immunotherapies might enhance the therapeutic effect.

### IL-P60_750_ in combination with a-PD-1 induced total tumor regression in all treated mice

**Fig. 6:**
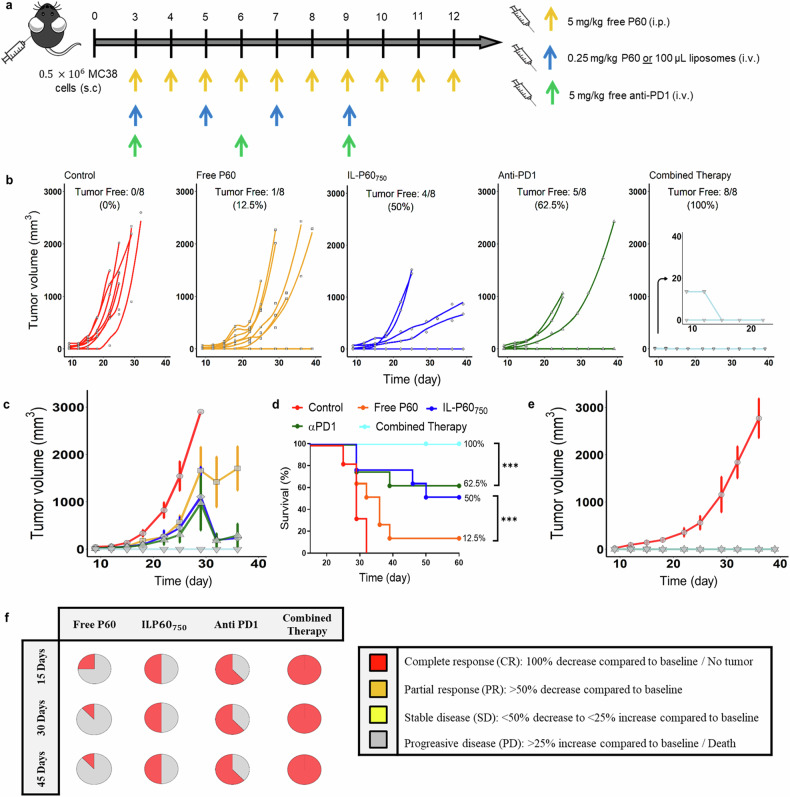
Antitumor in-vivo response measured in MC38 tumor-bearing mice. **a** Experimental design scheme; (**b**) individual tumor growth kinetics induced by the different treatments; (**c**) time profiles of tumor growth computed as the mean ± SEM for each group of treatment; (**d**) long-rank survival curves; (**e**) tumor growth kinetics in MC38 re-challenged mice (blue line) and control mice (red line); (**f**) tumor growth evolution for the different groups of treatment based on clinical RECIST criteria49 (*** *P* value < 0.001).

The antitumor effect of P60, free and encapsulated, was evaluated in combination with the immune checkpoint inhibitor a-PD-1. Total tumor regression induced by a-PD-1 (62.5%) in monotherapy was significantly (*P* = 0.0005) higher than the one induced by P60 monotherapy (12.5% for free and 50% for IL-P60_750_) at day 60 (Fig. 6b). However, the a-PD1 and IL-P60_750_ combination was more effective, achieving tumor rejection in 100% of mice at day 15 after treatment (Fig. 6c). Survival curves together with the plot corresponding to RECIST criteria show that this rapid initial effect was maintained after the end of treatment (Fig. 6d, f). Therefore, this combination supports the association of different mechanisms to achieve a higher antitumor effect than that observed for the monotherapies.

Moreover, cured mice remained tumor-free after re-challenge with MC38 cells (Fig. 6e), indicating that IL-P60_750_ was able to induce immune memory response.

No toxicities were associated with the treatments, supporting the safety of the formulation (Supplementary material Table S2 and Figs. S5, S6)

### IL-P60_750_ combined with a-PD-1 did not enhance efficacy in LLCOVA tumor model

**Fig. 7:**
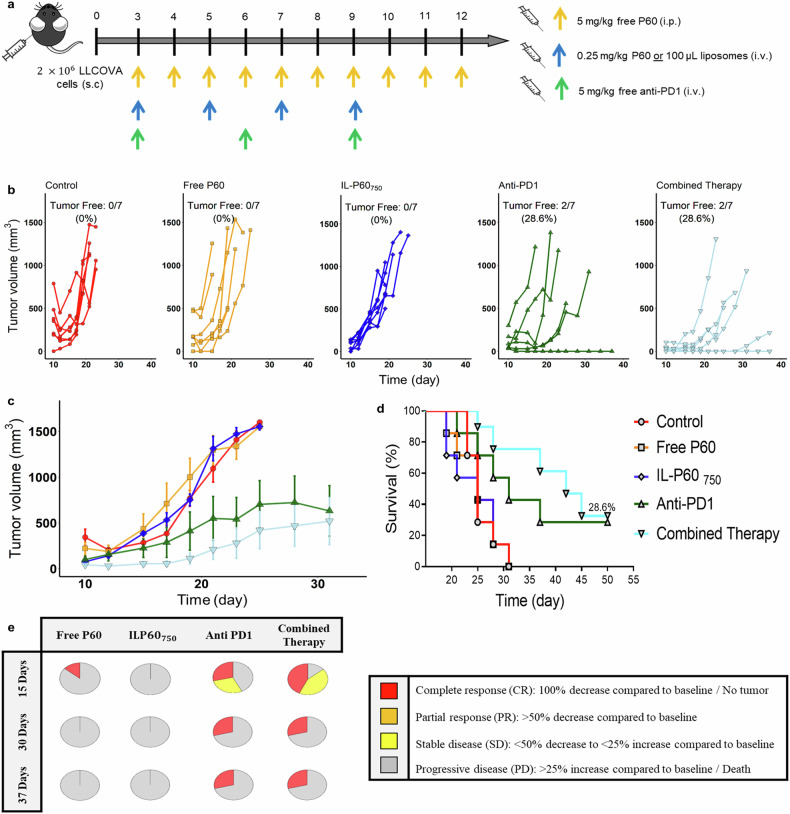
Antitumor in-vivo response measured in LLCOVA tumor-bearing mice. **a** Experimental design scheme; (**b**) individual tumor growth kinetics induced by the different treatments; (**c**) time profiles of tumor growth computed as the mean ± SEM for each group of treatment; (**d**) long-rank survival curves; (**e**) tumor growth evolution for the different groups of treatment based on clinical RECIST criteria [48].

The combined therapy was also evaluated in LLCOVA tumor model. In this model, neither free P60 nor IL-P60_750_ exerted a significant effect on tumor growth compared to the control group (Fig. 7b, *P* = 0.067). Thus, tumor growth profiles after the IL-P60_750_ and a-PD-1 combination, showed similar behavior to that induced by a-PD-1 monotherapy, although a slight delay in tumor growth could be observed over the first 10 d (Fig. 7c). Nevertheless, the rapid growth of tumors regardless of treatment, highlights the poor activity of P60 and a-CD25 in this tumor model, as shown the survival curves and the plot representing the RECIST criteria (Fig. 7d, e). This result might suggest the presence of other immune resistance mechanisms more relevant than Tregs in LLCOVA model.

### IL-P60_750_ inhibits human Treg proliferation and activity

**Fig. 8:**
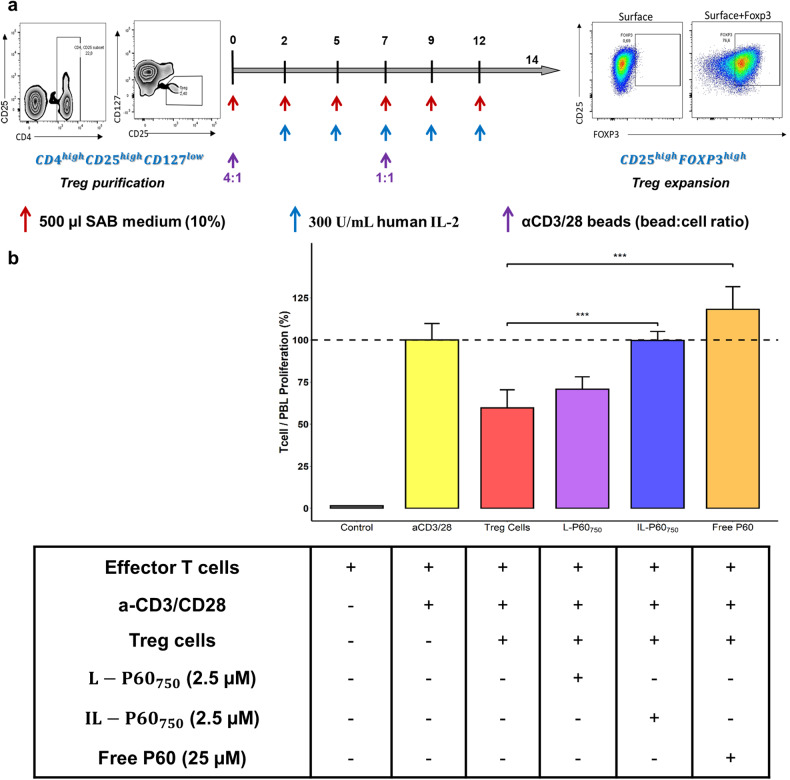
Ex-vivo study using a co-culture of human Treg and CD8^+^ T cells to evaluate the effect of P60 nontargeted and IL-P60_750_ formulated with human anti-CD25 Fab’. **a** Procedure to select and expand Treg collected from umbilical cord samples; (**b**) effect of Treg and P60, free and encapsulated (L-P60_750_ / ILP60_750_), on effector CD8^+^ T cells proliferation, stimulated with a-CD3/CD28. Bars represent the average of six independent samples with the standard deviation. (** *P* value < 0.01, *** *P* value < 0.001).

The potential translation of this novel formulation has been explored using a co-culture of human Tregs and effector CD8^+^ T cells stimulated with a-CD3/CD28 magnetic beads. Tregs induced a 30% reduction in effector CD8^+^ T cell proliferation (Fig. 8). This effect was totally overcome by the exposure to human IL-P60_750_ that allowed the recovery of CD8^+^ T cells to basal levels, 99.67% ± 5.33%, (*P* = 1.402 × 10^−6^), at a concentration 10 times lower (IL-P60_750_ 2.5 μM) than for free P60 (25 μM). In contrast, non-targeted liposomes were not able to overcome the inhibitory activity of Treg cells.

## Discussion

An efficient inactivation of Tregs remains an important challenge in cancer immunotherapy, especially for those tumors characterized by the high infiltration of these cells into tumor microenvironment. In this scenario, P60 has demonstrated the ability to modulate Treg activity [9, 32]. However, in-vivo instability and poor target specificity are important shortcomings to achieve translational relevance that we have addressed in this study.

Hence, we have developed liposomes for P60 encapsulation using the lipid film hydration method. The low encapsulation efficiency (20%) was improved with the urea chaotropic method, reaching >50% of peptide encapsulation. This change might be attributed to the urea binding to amide units and side chains of P60 via hydrogen bonds, increasing the stability and solubility of the peptide [10, 33]. Similar effect in the encapsulation has been reported by other authors for highly hydrophobic peptides such as p5 using 7 M of urea. In this case both, 3.5 and 7 M of urea led to obtain liposomes with similar characteristics, selecting this lower concentration to reduce a possible cell toxicity of urea [34].

On the other hand, PEG, commonly used to formulate liposomes, prolongs the circulation time of these formulations by modifying the corona effect or protein adsorption [35, 36]. In particular, DSPE-PEG_2000_ is one of the reagents included in clinical approved PEGylated liposomes, such as Doxil [37]. However, in the particular case of targeted liposomes, the polymeric layer coating the lipids might embed ligands conjugated to the end of the functionalized polymer. This reduces receptor recognition, targeting ability and selective cargo delivery [35]. This hindrance of ligand-receptor binding has been related to the brush conformation adopted by PEG at a specific density ( ≥ 5%) similar to that used in this study [35]. To address this limitation, several strategies have been investigated, notably the combination of PEGs of different lengths. Nevertheless, this interesting approach has not yet been fully explored [35, 38]. Therefore, one of the aims of this work was the preparation of two targeted formulations encapsulating P60 and using the post-insertion method that combines DSPE-PEG_2000_-Fab´CD25 micelles with conventional DSPE-PEG_2000_ liposomes or novel DSPE-PEG_750_ liposomes providing then, ILs: (i) ILs formulated with DSPE-PEG_2000_ only and (ii) ILs combining DSPE-PEG_750_ and DSPE-PEG_2000_ of micelles. The coating thickness is the main difference between these two targeted formulations, a characteristic that may help to study the behavior of ligands regarding the receptor recognition (Fig. 1). P60 encapsulation efficiency as well as other physicochemical characteristics of liposomes, non-targeted and targeted, were not affected by the type of PEG. Shorter polymer chains (DSPE-PEG_750_), associated with a higher permeability coefficient, can reduce slightly the in-vitro and in-vivo stability [35]. However, in this work, this DSPE-PEG_750_ formulation showed reasonable stability for almost one month, with a release lower than 20% during the first 24 h in presence of serum, a result that supported the intravenous administration of formulations.

Notably, ex vivo results demonstrated that both IL formulations (IL-PEG_2000_ and IL-PEG_750_) bound selectively to CD25, although the uptake and internalization was 2 times higher for IL formulated with shorter chain lengths, suggesting a better ligand exposure at the surface of liposomes. Therefore, we hypothesized that DSPE-PEG_750_ decreases the steric hindrance and allows a more flexible orientation of ligands, improving receptor recognition and promoting higher liposome binding and internalization. To our knowledge, this is the first targeting formulation that combines in the same formulation DSPE-PEG_750_ and DSPE-PEG_2000_, to improve the extent and efficacy of the targeting moiety.

These encouraging findings led to select this formulation for in-vivo experiments. Notably chosen cell lines showed different results as expected, likely influenced by the different role or weight that Tregs may play in tumor development within each tumor model. In fact, in vivo administration of IL-P60_750_ faces the challenge of bypassing circulating CD25^+^ cells such as NK, CD8^+^ T cells and Tregs before reaching the TME [39]. Interestingly, Foxp3 is not only expressed on Tregs as also CD8^+^ effector T cells can transiently express Foxp3 after TCR stimulation. This transient expression of Foxp3 could act as a feedback mechanism to quickly turn off the activation state of tumor-infiltrating effector T cells, contributing to the loss of antitumor responses [40–42]. In this particular case, IL-P60_750_ can bind to CD8^+^ effector T cells and then, it might be able to re-activate these cells, contributing to the antitumor effect and inhibiting the activity of Treg population. Nevertheless, ILs also bind to CD25^+^ Foxp3^-^ cells (i.e., NK cells), which might capture nanoparticles, reducing the availability of IL for Treg cells and reducing therefore, the therapeutic effect of the encapsulated P60. In fact, ILs uptake by NK cells was observed in both assayed tumor models; however, the impact was higher in LLCOVA tumors. This fact associated with the poor therapeutic effect of P60, free and encapsulated, suggest that in this model Treg cells did not play the most relevant immunoresistance role. In contrast, the uptake of IL-P60_750_ by the CD25^+^ cells present in systemic circulation did not compromise the therapeutic effect in the MC38 murine model. In fact, the higher antitumor response induced by ILs at a dose 50 times lower than for free P60, suggests that ILs are able to modulate several mechanisms: (i) CD25 targeting increases Treg selectivity and P60 bioavailability; (ii) CD25 targeting decreases CD25 availability and hampers IL-2 binding (cytokine responsible for the expansion and survival of Treg cells), and (iii) P60 impairs Foxp3 inhibitory activity [16]. Besides, the higher therapeutic effect of IL-P60_750_ was associated with higher activation of effector CD8^+^ T cells in lymph nodes, spleen and tumor. In tumor, IFNγ levels and Granzyme B expression were also increased, a fact that might indicate a shift to a pro-inflammatory microenvironment derived from the inactivation of Tregs by ILs [43, 44]. It is interesting to highlight that IL-P60_750_, on the contrary to other immunostimulatory methods based on Treg depletion, did not decrease the number of Tregs, which might reduce the potential risk of collateral exacerbation of autoimmunity [45].

Furthermore, IL-P60_750_ combined with a-PD-1 led to cure 100% of the mice, a finding similar to that reported by Arce Vargas and coworkers in the MC38 model combining a-CD25 and a-PD-1 [46]. The potentiation of the response derived from this combination can be explained by the removal of two main brakes, one exerted by Tregs on effector cells, which is inhibited by IL-P60_750_, and the second, by the re-activation of exhausted effector cells mediated by a-PD-1 (hampering the PD-1/PD-L1 binding). Moreover, it is noteworthy that IL-P60_750_ induced antitumor immune memory, a phenomenon associated with an efficient activation of an adaptive immune response [47].

The limited response achieved by IL-P60_750_ in the LLCOVA tumor could be linked to the existence of additional immune resistance mechanisms beyond Treg presence. These divergent responses observed in different tumor types underscore the potential value of stratifying tumors according to their unique tumor microenvironment characteristics, which could aid in the personalized selection of immunotherapies.

Finally, as a proof-of-concept for a potential clinical translation, IL-P60_750_ was easily adapted for human CD25 targeting. This modification did not change liposome characteristics, demonstrating the robustness of the developed methodology and the dynamic properties of the liposomes. In this study, this novel targeted nanoplatform also inhibited the activity of human Tregs, giving the opportunity to recover efficiently the proliferative activity of effector CD8^+^ T cells.

## Conclusion

All results together have demonstrated that it is feasible to prepare a homogeneous population of CD25-targeted P60 liposomes able to bind selectively to Tregs. These formulations increase the stability and intracellular delivery of P60, promoting an efficient antitumor adaptive immune response, inducing a pro-inflammatory microenvironment.

In addition, the combination of CD25-targeted P60 liposomes with PD-1 immune checkpoint inhibitors enhances antitumor efficacy, showing promise for improving current immunotherapies.

Overall, the combination of different PEG chain lengths allowed us to develop a flexible targeted nanoplatform able to encapsulate a broad range of therapeutic molecules.

## Supplementary information

Supplementary Figure S1

Supplementary Figure S2

Supplementary Figure S3

Supplementary Figure S4

Supplementary Figure S5

Supplementary Figure S6

Supplementary material

Supplementary Table S1

Supplementary Table S2

## Data Availability

Data generated in this study are available upon reasonable request from the corresponding authors.

## References

[1] Park K, Veena MS, Shin DS. Key players of the immunosuppressive tumor microenvironment and emerging therapeutic strategies. Front Cell Dev Biol. 2022;10:1–24. 10.3389/fcell.2022.830208 PMC8957227 35345849

[2] Chen BJ, Zhao JW, Zhang DH, Zheng AH, Wu GQ. Immunotherapy of cancer by targeting regulatory T cells. Int Immunopharmacol. 2022;104:108469. 35008005 10.1016/j.intimp.2021.108469

[3] Tanaka A, Sakaguchi S. Regulatory T cells in cancer immunotherapy. Cell Res. 2017;27:109–18. 27995907 10.1038/cr.2016.151 PMC5223231

[4] Ohue Y, Nishikawa H. Regulatory T (Treg) cells in cancer: Can Treg cells be a new therapeutic target? Cancer Sci. 2019;110:2080–9. 31102428 10.1111/cas.14069 PMC6609813

[5] Savage PA, Malchow S, Leventhal DS. Basic principles of tumor-associated regulatory T cell biology. Trends Immunol. 2013;34:33–40. 22999714 10.1016/j.it.2012.08.005 PMC3534814

[6] Tanaka A, Sakaguchi S. Targeting Treg cells in cancer immunotherapy. Eur J Immunol. 2019;49:1140–6. 31257581 10.1002/eji.201847659

[7] Colombo MP, Piconese S. Regulatory T-cell inhibition versus depletion: the right choice in cancer immunotherapy. Nat Rev Cancer. 2007;7:880–7. 17957190 10.1038/nrc2250

[8] Lozano T, Casares N, Lasarte JJ. Searching for the Achilles heel of FOXP3. Front Oncol. 2013;3:1–9. 24350059 10.3389/fonc.2013.00294 PMC3847665

[9] Casares N, Rudilla F, Arribillaga L, Llopiz D, Riezu-Boj J, Lozano T, et al. A peptide inhibitor of FOXP3 impairs regulatory T cell activity and improves vaccine efficacy in mice. J Immunol. 2010;185:5150–9. 20870946 10.4049/jimmunol.1001114

[10] Yazdani M, Amir S, Badiee A, Shariat S, Mansourian M, Arabi L, et al. Stimulation of tumor-specific immunity by p5 HER-2/neu generated peptide encapsulated in nano-liposomes with high phase transition temperature phospholipids. Curr Drug Deliv. 2016;14:492–502. 10.2174/1567201813666160712164149 27411392

[11] van Elk M, Murphy B, Eufrásio-da-Silva T, O’Reilly D, Vermonden T, Hennink W, et al. Nanomedicines for advanced cancer treatments: Transitioning towards responsive systems. Int J Pharmacol. 2016;515:132–64. 10.1016/j.ijpharm.2016.10.013 27725268

[12] Fang J, Nakamura H, Maeda H. The EPR effect: Unique features of tumor blood vessels for drug delivery, factors involved, and limitations and augmentation of the effect. Adv Drug Deliv Rev. 2011;63:136–51. 20441782 10.1016/j.addr.2010.04.009

[13] Merino M, Zalba S, Garrido MJ. Immunoliposomes in clinical oncology: State of the art and future perspectives. J Control Release. 2018;275:162–76. 29448116 10.1016/j.jconrel.2018.02.015

[14] Becker C, Göpferich A. Passive and active drug targeting: drug delivery to tumors as an example. Tissue Eng. 2007;197:3–53. 10.1007/978-3-642-00477-3_1 20217525

[15] Eloy JO, Petrilli R, Trevizan LNF, Chorilli M. Immunoliposomes: a review on functionalization strategies and targets for drug delivery. Colloids Surf B Biointerfaces. 2017;159:454–67. 28837895 10.1016/j.colsurfb.2017.07.085

[16] Damoiseaux J. The IL-2–IL-2 receptor pathway in health and disease: the role of the soluble IL-2 receptor. Clin Immunol. 2020;218:108515. 32619646 10.1016/j.clim.2020.108515

[17] Hak S, Helgesen E, Hektoen H, Huuse E, Jarzyna A, Mulder W, et al. The effect of nanoparticle polyethylene glycol surface density on ligand-directed tumor targeting studied in vivo by dual modality imaging. ACS Nano. 2012;6:5648–58. 22671719 10.1021/nn301630n PMC3389615

[18] Merino M, Lozano T, Casares N, Lana H, Troconiz I, ten Hagen T, et al. Dual activity of PD-L1 targeted doxorubicin immunoliposomes promoted an enhanced efficacy of the antitumor immune response in melanoma murine model. J Nanobiotechnol. 2021;19:1–15. 10.1186/s12951-021-00846-z PMC8042980 33849551

[19] Rothdiener M, Beuttler J, Messerschmidt SKE, Kontermann RE. Antibody targeting of nanoparticles to tumor-specific receptors: immunoliposomes. Methods Mol Biol 2010;624:295–308. 20217604 10.1007/978-1-60761-609-2_20

[20] Lozano T, Gorraiz M, Lasarte-Cía A, Ruiz M, Rabal O, Oyarzabal J, et al. Blockage of FOXP3 transcription factor dimerization and FOXP3/AML1 interaction inhibits T regulatory cell activity: sequence optimization of a peptide inhibitor. Oncotarget. 2017;8:71709–24. 29069740 10.18632/oncotarget.17845 PMC5641083

[21] Xiang B, Cao DY. Preparation of drug liposomes by thin-film hydration and homogenization. Liposome-Based Drug Deliv Syst. 2018;1–11. 10.1007/978-3-662-49231-4_2-1.

[22] Alipour Talesh G, Ebrahimi Z, Badiee A, Mansourian M, Attar H, Arabi L, et al. Poly (I: C)-DOTAP cationic nanoliposome containing multi-epitope HER2-derived peptide promotes vaccine-elicited anti-tumor immunity in a murine model. Immunol Lett. 2016;176:57–64. 27260485 10.1016/j.imlet.2016.05.016

[23] Merino M, Contreras A, Casares N, Troconiz I, Ten Hagen T, Berraondo P, et al. A new immune-nanoplatform for promoting adaptive antitumor immune response. Nanomed Nanotechnol Biol Med. 2019;17:13–25. 10.1016/j.nano.2018.12.016 30654186

[24] Rouser G, Fleischer S, Yamamoto A. Two dimensional thin layer chromatographic separation of polar lipids and determination of phospholipids by phosphorus analysis of spots. Lipids. 1970;5:494–6. 5483450 10.1007/BF02531316

[25] Hussain MT, Forbes N, Perrie Y. Comparative analysis of protein quantification methods for the rapid determination of protein loading in liposomal formulations. Pharmaceutics. 2019;11:9–11. 10.3390/pharmaceutics11010039 PMC6358724 30669330

[26] Xu X, Khan MA, Burgess DJ. A two-stage reverse dialysis in vitro dissolution testing method for passive targeted liposomes. Int J Pharmacol. 2012;426:211–8. 10.1016/j.ijpharm.2012.01.030 22301423

[27] Lozano T, Casares N, Martil-Otal C, Anega B, Gorraiz M, Parker J, et al. Searching for peptide inhibitors of t regulatory cell activity by targeting specific domains of foxp3 transcription factor. Biomedicines. 2021;9:1–20. 10.3390/biomedicines9020197 PMC7922534 33671179

[28] Lim EL, Cugliandolo F, Rosner D, Gyori D, Roychoudhuri R, Okkenhaug K. Phosphoinositide 3-kinase δ inhibition promotes antitumor responses but antagonizes checkpoint inhibitors. JCI Insight. 2018;3:e120626. 29875319 10.1172/jci.insight.120626 PMC6124416

[29] Chen L, Huang H, Zheng X, Li Y, Chen J, Tan B, et al. IL1R2 increases regulatory T cell population in the tumor microenvironment by enhancing MHC-II expression on cancer-associated fibroblasts. J Immunother Cancer. 2022;10:e004585.

[30] Saeed M, Zalba S, Seynhaeve ALB, Debets R, Ten Hagen TLM. Liposomes targeted to MHC-restricted antigen improve drug delivery and antimelanoma response. Int J Nanomed. 2019;14:2069–89. 10.2147/IJN.S190736 PMC6440454 30988609

[31] Lasarte-Cia A, Lozano T, Pérez-González M, Gorraiz M, Iribarren K, Hervás-Stubbs S, et al. Immunomodulatory properties of carvone inhalation and its effects on contextual fear memory in mice. Front Immunol. 2018;9:1–12. 29422905 10.3389/fimmu.2018.00068 PMC5788902

[32] Moreno MA, Florencia M, Imsen M, Asad A, Bal de Kier E, Casares N, et al. Therapeutic blockade of Foxp3 in experimental breast cancer models. Breast Cancer Res Treat. 2017;166:393–405. 28756536 10.1007/s10549-017-4414-2

[33] Canchi DR, García AE. Cosolvent effects on protein stability. Annu Rev Phys Chem. 2013;64:273–93. 23298246 10.1146/annurev-physchem-040412-110156

[34] Wang N, Wang Y, Shi R, Lin Y, Jiang X, Feng Y, et al. The photodynamic/photothermal synergistic therapeutic effect of BODIPY-I-35 liposomes with urea. Photodiagnosis Photodyn Ther. 2022;37:102723. 35032702 10.1016/j.pdpdt.2022.102723

[35] Zalba S, ten Hagen TLM, Burgui C, Garrido MJ. Stealth nanoparticles in oncology: facing the PEG dilemma. J Control Release. 2022;351:22–36. 36087801 10.1016/j.jconrel.2022.09.002

[36] Lee H, Larson RG. Adsorption of plasma proteins onto PEGylated lipid bilayers: the effect of PEG size and grafting density. Biomacromolecules. 2016;17:1757–65. 27046506 10.1021/acs.biomac.6b00146

[37] Barenholz Y. Doxil® - The first FDA-approved nano-drug: lessons learned. J Control Release. 2012;160:117–34. 22484195 10.1016/j.jconrel.2012.03.020

[38] Guo B, Xu D, Liu X, Yi J. Enzymatic synthesis and in vitro evaluation of folate-functionalized liposomes. Drug Des Devel Ther 2017;11:1839–47. 28684902 10.2147/DDDT.S132841 PMC5484511

[39] Spolski R, Li P, Leonard WJ. Biology and regulation of IL-2: from molecular mechanisms to human therapy. Nat Rev Immunol. 2018;18:648–59. 30089912 10.1038/s41577-018-0046-y

[40] Machicote A, Belén S, Baz P, Billordo LA, Fainboim L. Human CD8^+^ HLA-DR^+^ regulatory T cells, similarly to classical CD4^+^Foxp3^+^cells, suppress immune responses via PD-1/PD-L1 axis. Front Immunol. 2018;9:1–13. 30555473 10.3389/fimmu.2018.02788 PMC6281883

[41] Heeren AM, Rotman J, Stam A, Pocorni N, Gassama A, Samuels S, et al. Efficacy of PD-1 blockade in cervical cancer is related to a CD8^+^FoxP3^+^ CD25^+^ T-cell subset with operational effector functions despite high immune checkpoint levels. J Immunother Cancer. 2019;7:1–14. 30755279 10.1186/s40425-019-0526-z PMC6373123

[42] Lozano T, Conde E, Martín-Otal C, Navarro F, Lasarte-Cia A, Nasrallah R, et al. TCR-induced FOXP3 expression by CD8^+^ T cells impairs their anti-tumor activity. Cancer Lett. 2022;528:45–58. 34973390 10.1016/j.canlet.2021.12.030

[43] Semmrich M, Marchand J, Fend L, Rehn M, Remy C, Homkvist P, et al. Vectorized Treg-depleting αcTLA-4 elicits antigen cross-presentation and CD8^+^ T cell immunity to reject cold’ tumors. J Immunother Cancer. 2022;10:1–14. 10.1136/jitc-2021-003488 PMC8783833 35058324

[44] Liang S, Zheng D, Liu X, Mei X, Zhou C, Xiao C, et al. BAT6026, a novel anti-OX40 antibody with enhanced antibody dependent cellular cytotoxicity effect for cancer immunotherapy. Front Oncol 2023;13:1–11. 10.3389/fonc.2023.1211759 PMC10421724 37576888

[45] Sakaguchi S, Sakaguchi N, Asano M, Itoh M, Toda M. Immunologic self-tolerance maintained by activated T cells expressing IL-2 receptor alpha-chains (CD25). Breakdown of a single mechanism of self-tolerance causes various autoimmune diseases. J Immunol. 2011;186:3808–21. 21422251

[46] Arce Vargas F, Furness A, Solomon I, Joshi K, Mekkaoui L, Lesko M, et al. Fc-optimized anti-CD25 depletes tumor-infiltrating regulatory T cells and synergizes with PD-1 blockade to eradicate established tumors. Immunity. 2017;46:577–86. 28410988 10.1016/j.immuni.2017.03.013 PMC5437702

[47] Barnaba V. T cell memory in infection, cancer, and autoimmunity. Front Immunol 2022;12:811968. 35069600 10.3389/fimmu.2021.811968 PMC8771143

[48] Eisenhauer E, Therasse P, Bogaets J, Schwartz L, Sargent D, Ford R, et al. New response evaluation criteria in solid tumours: Revised RECIST guideline (version 1.1). Eur J Cancer. 2009;45:228–47. 19097774 10.1016/j.ejca.2008.10.026

