# Supplementary figures and images for: Foxp3 inhibitory peptide encapsulated in a novel CD25-targeted nanoliposome promotes efficient tumor regression in mice
Source: Acta Pharmacol Sin. 2024 Jul 29;46(1):171–83. doi: 10.1038/s41401-024-01338-0 (PMC11695603; doi:10.1038/s41401-024-01338-0)

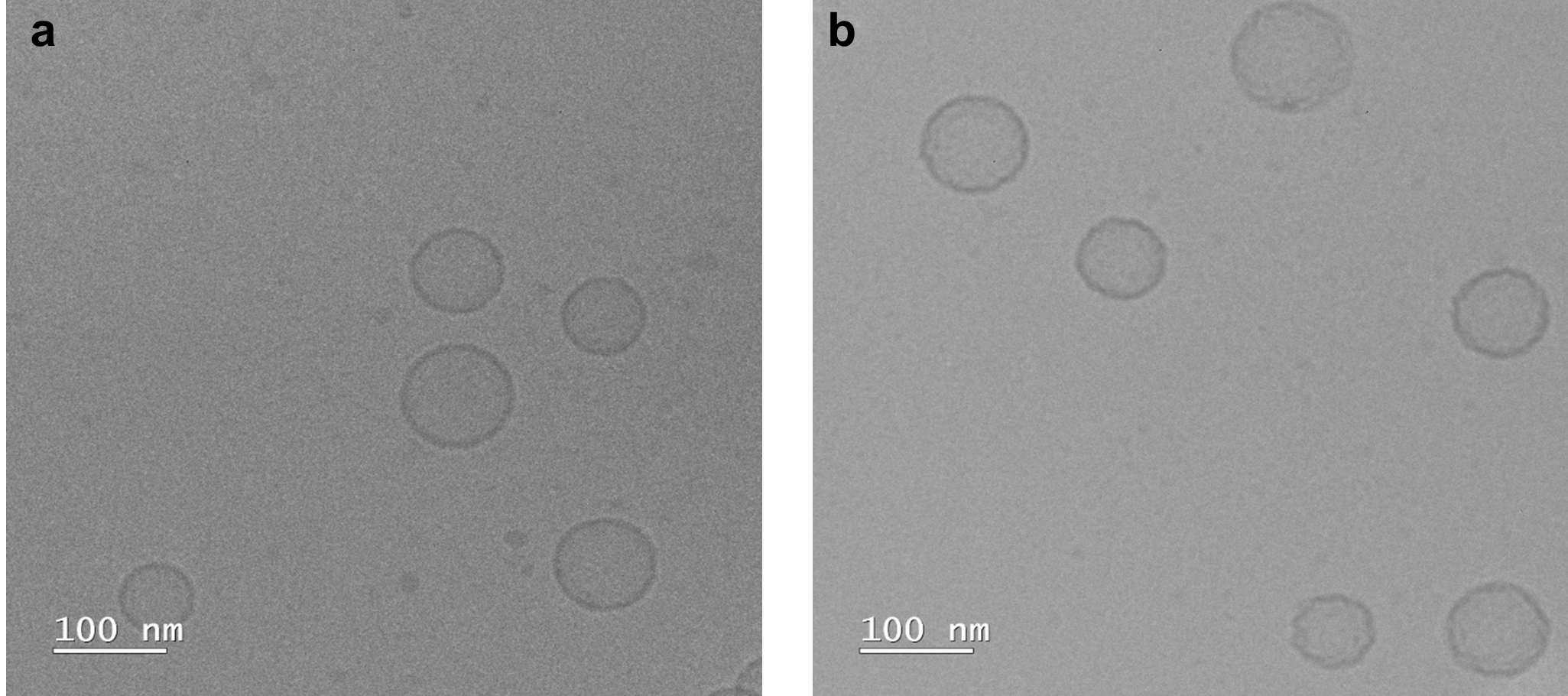

Supplement: Supplementary file 1 — Supplementary Figure S1 [file 41401_2024_1338_MOESM1_ESM.tif]

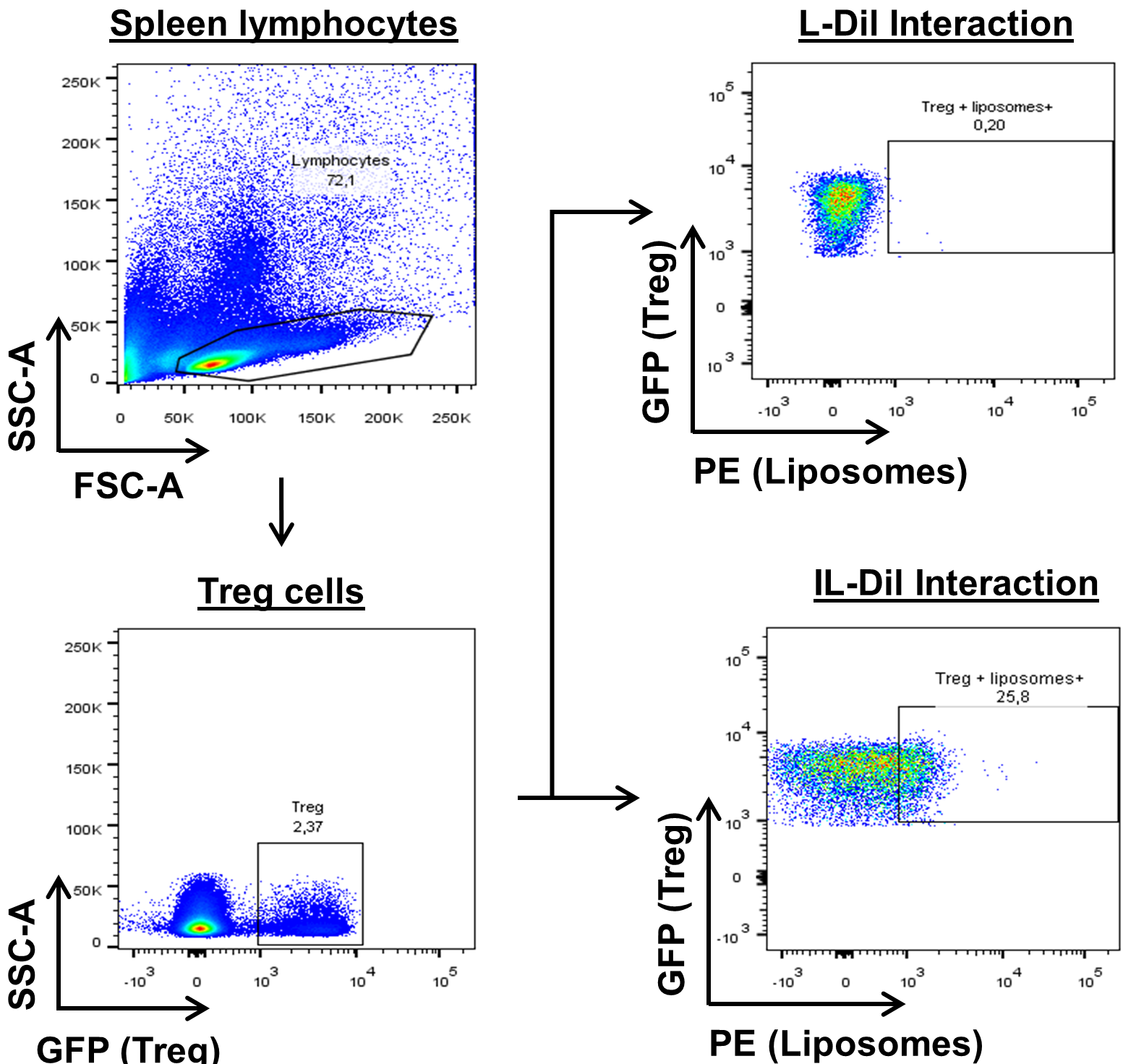

Supplement: Supplementary file 2 — Supplementary Figure S2 [file 41401_2024_1338_MOESM2_ESM.tif]

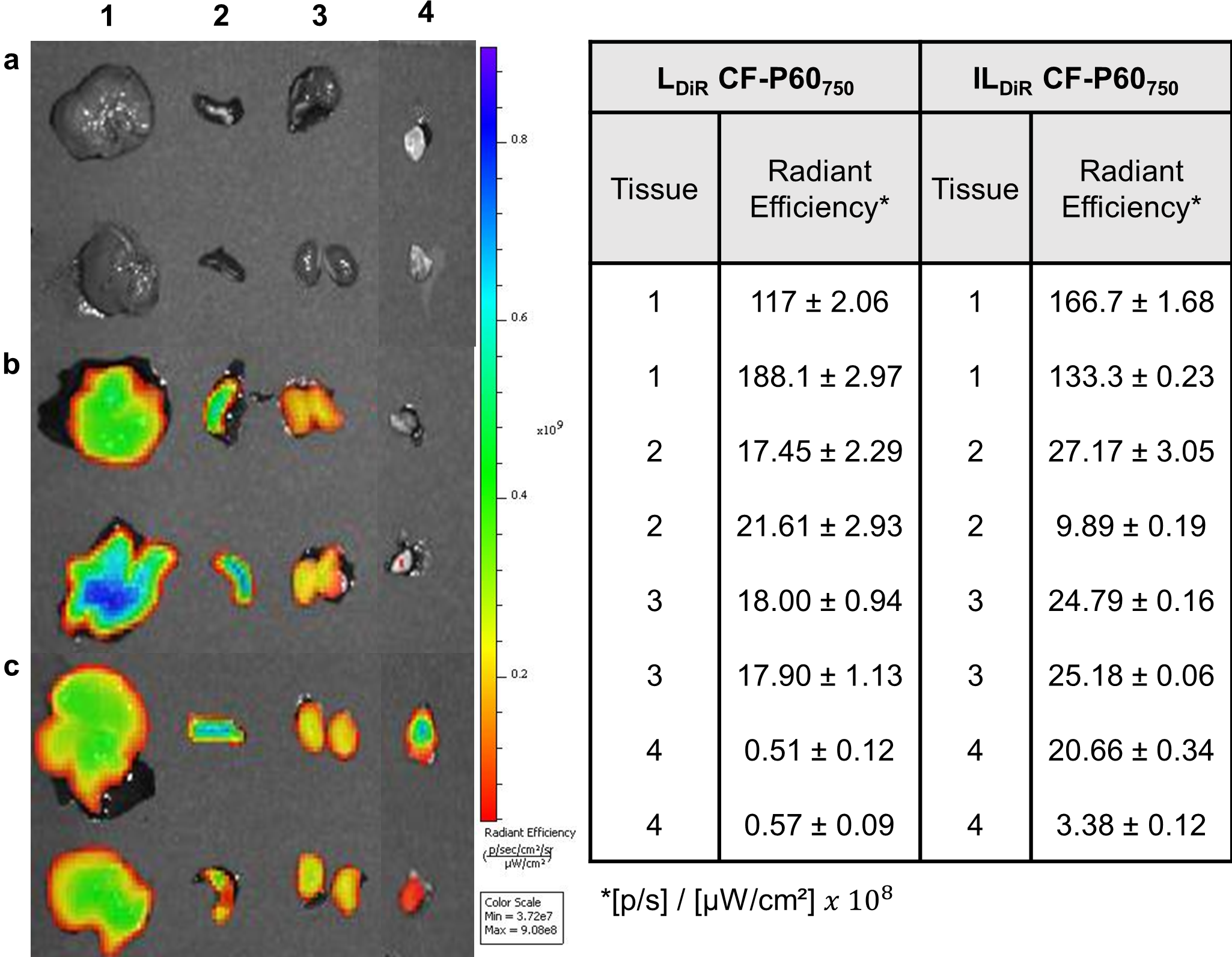

Supplement: Supplementary file 3 — Supplementary Figure S3 [file 41401_2024_1338_MOESM3_ESM.tif]

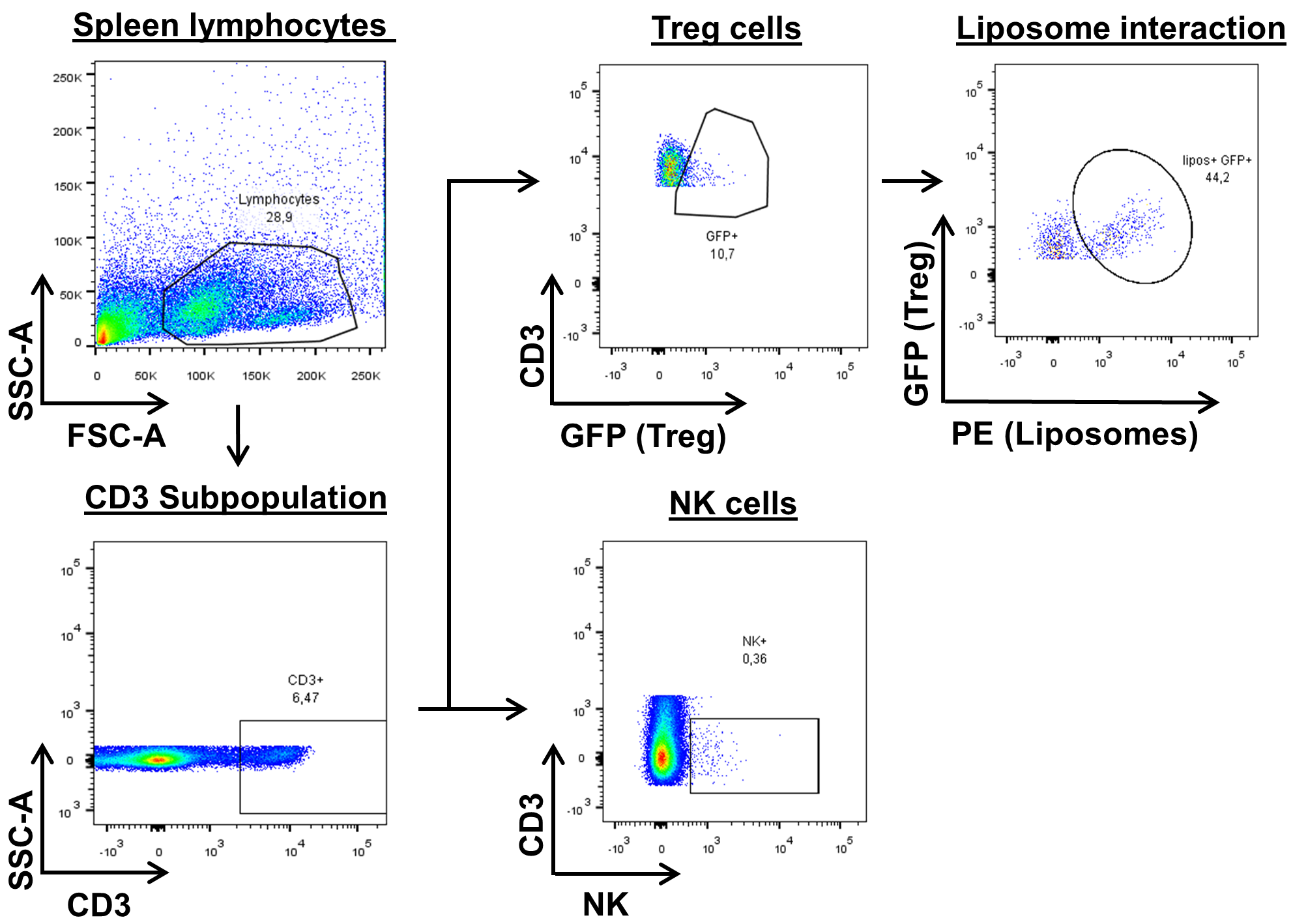

Supplement: Supplementary file 4 — Supplementary Figure S4 [file 41401_2024_1338_MOESM4_ESM.tif]

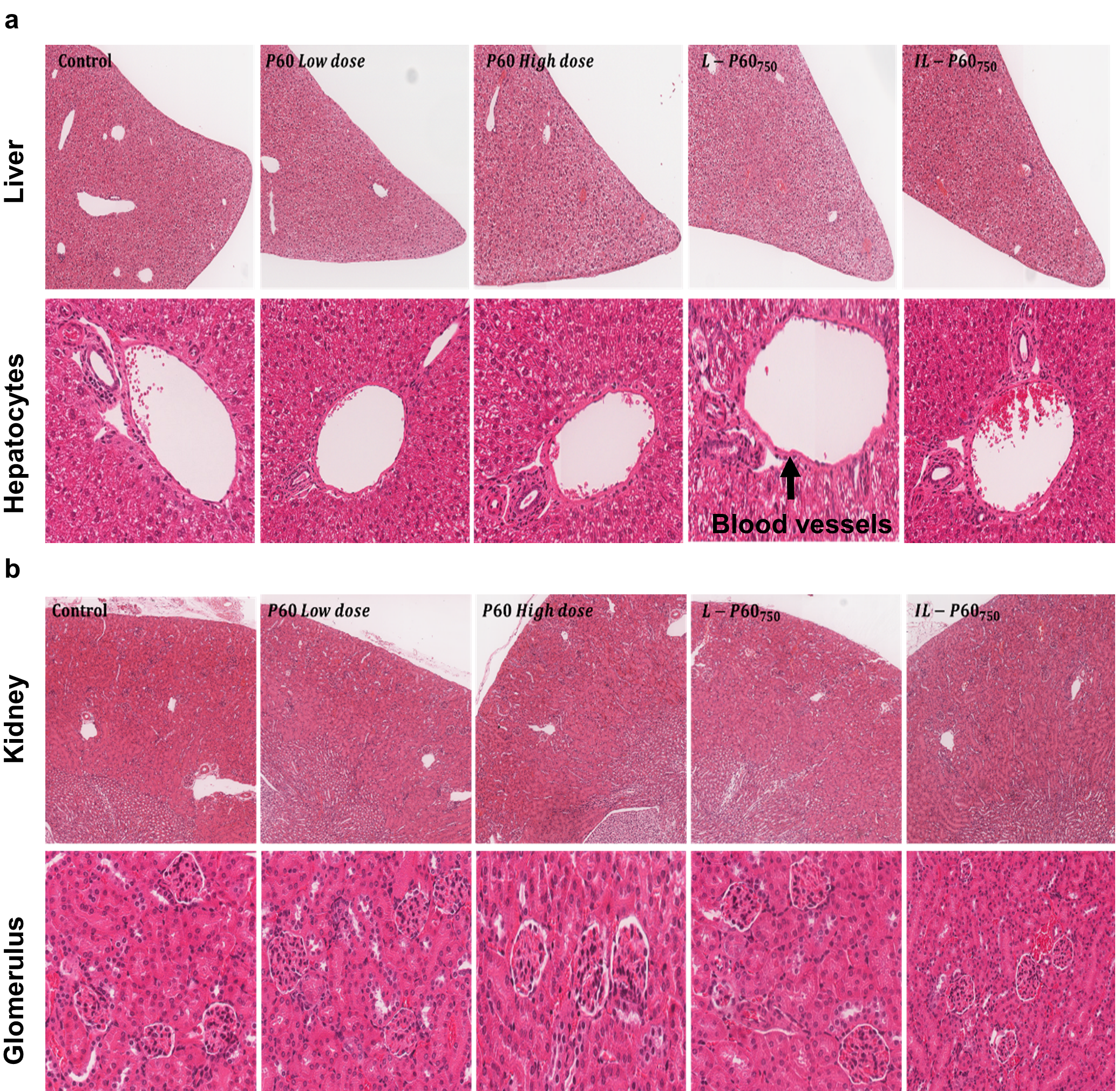

Supplement: Supplementary file 5 — Supplementary Figure S5 [file 41401_2024_1338_MOESM5_ESM.tif]

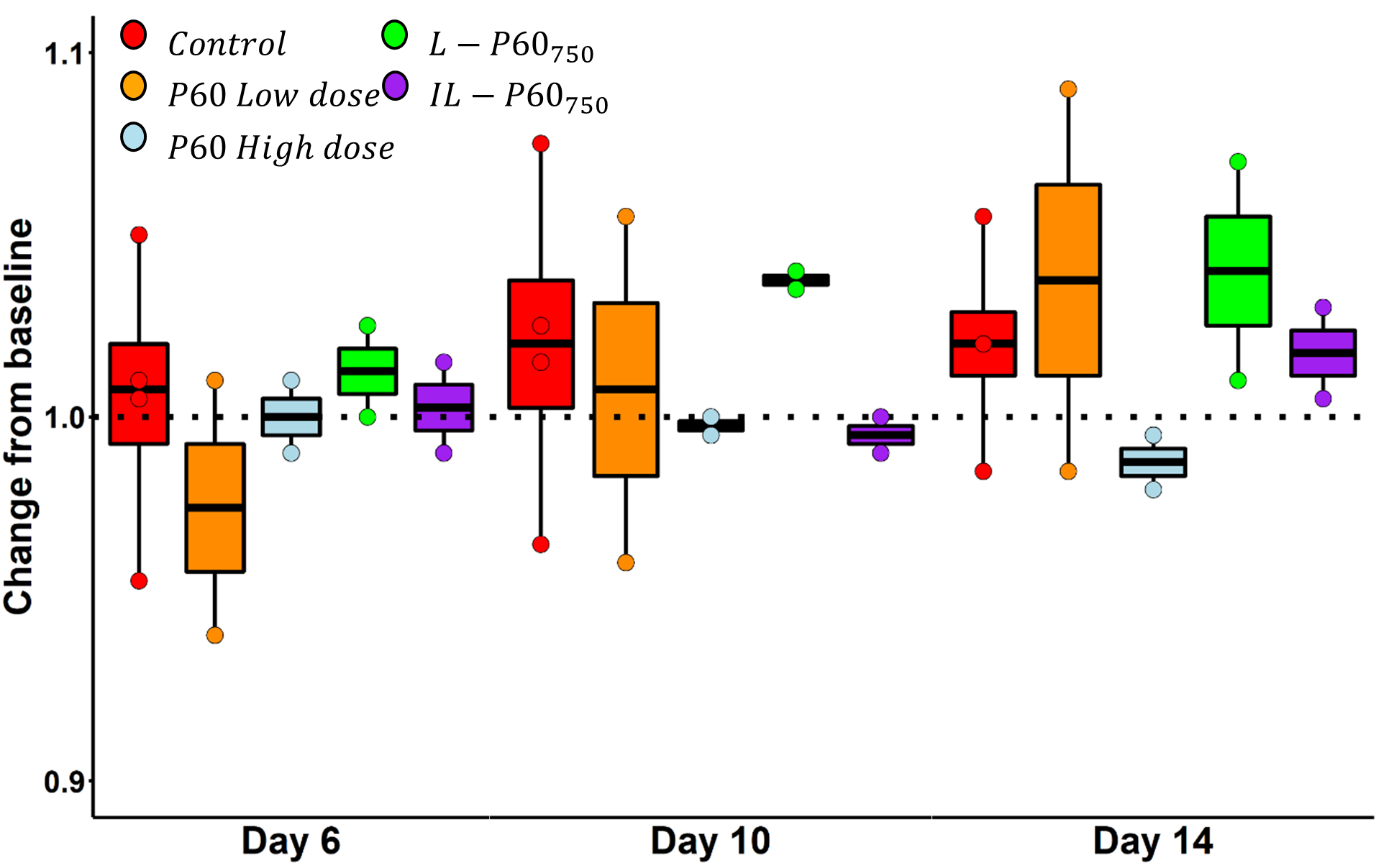

Supplement: Supplementary file 6 — Supplementary Figure S6 [file 41401_2024_1338_MOESM6_ESM.tif]
