# Supplementary material for: Foxp3 inhibitory peptide encapsulated in a novel CD25-targeted nanoliposome promotes efficient tumor regression in mice
Source: Acta Pharmacol Sin. 2024 Jul 29;46(1):171–83. doi: 10.1038/s41401-024-01338-0 (PMC11695603; doi:10.1038/s41401-024-01338-0)
Supplement: Supplementary file 7 — Supplementary material [file 41401_2024_1338_MOESM7_ESM.docx]

**Supplementary material.**

**Cryo-TEM images of formulations***.*

Liposomal formulations, non-targeted and targeted, were analyzed using cryo-Transmission Electron Microscopy (cryo-TEM). Samples were equilibrated from 4º C to RT, in order to get the lipids closer to their liquid state and avoid shape artifacts. Quanytifoil R2/1 200mesh Copper grids (SPI supplies, USA) were glow discharged (air plasma) for 2.5 min and immediately mounted on an FEI Vitrobot Mark IV (Thermo Fisher Scientific, USA) at 25º C and 100% humidity. Then, 3 μL sample was applied and blotted for 1 min at force 0 and left it drain for 0.5 s before automated shooting into liquid ethane. Samples were analized immediately with a Jeol JEM 1400 plus (Jeol, Japan) operated at an accelerating voltage of 80kV equipped with a LaB6 source. Results are represented in Figure S1.

**In-vivo L-P60_750_ and IL-P60_750_ tumor uptake**

Tumor uptake of targeted and non-targeted liposomes was assayed using fluorescent formulations. These liposomes, formulated with DIR (a lipophilic, near-infrared fluorescent cyanine dye), were prepared with P60 conjugated with carboxifluorescein (CF-P60). Briefly, twelve C57B6/J mice (n = 4 mice/group) were sc inoculated with 5x10^5^ MC38 cells in 100 μL of PBS. A week later, when tumor reached ~ 50-80 mm^3^, mice were randomly divided into three groups: Group 1 or non-treated; Group 2 treated with Non-targeted or L_DiR_ CF-P60_750_ and Group 3 treated with Targeted liposomes or IL_DiR_ CF-P60_750_. Mice were analyzed by in-vivo imaging system (IVIS) and afterwards, liver, spleen, kidneys and tumors were collected to visualize the presence of liposomes in each group of treatment. Non-treated group was the background control*.*

Tumor accumulation of IL-P60 was much higher than that of L-P60 supporting the selective targeting and therefore, antitumor response observed in the MC38 tumor model. Results are represented in Figure S3.

**In-vivo toxicity assay**

A total of 12 C57B6/J female mice were subcutaneously inoculated with 5x10^5^ MC38 cells in 100 μl of PBS. Six days later, mice were randomly divided into five groups (n = 2-4 mice/group), according to the previous experimental design: control group (non-treated), free P60 i.v. at low dose (0.25 mg/Kg every 2 days for 4 doses), free P60 i.v. at high dose (5 mg/Kg/day for 10 doses), non-targeted i.p. P60 liposomes (LP60; 0.25 mg/Kg every 2 days for 4 doses), and targeted i.p. P60 liposomes (ILP60; 0.25 mg/Kg every 2 days for 4 doses).

Tumor growth and body weight were monitored every 2 days up until the end of the study corresponding to the end of treatments. Then, mice were sacrificed to collect blood samples and organs. Blood samples were evaluated using COBAS 6000 Analyzer Series (Roche, Switzerland), while organ tissues were stained with hematoxylin-eosin and analyzed using Aperio CS2 Digital pathology slide scanner (Leica, Spain). Results are represented in Table S2, and Figures S5 and S6.
