# Supplementary material for: Foxp3 inhibitory peptide encapsulated in a novel CD25-targeted nanoliposome promotes efficient tumor regression in mice
Source: Acta Pharmacol Sin. 2024 Jul 29;46(1):171–83. doi: 10.1038/s41401-024-01338-0 (PMC11695603; doi:10.1038/s41401-024-01338-0)
Supplement: Supplementary file 8 — Supplementary Table S1 [file 41401_2024_1338_MOESM8_ESM.docx]

**Table S1.** Reagents and antibodies employed for the cytometry analysis performed in the study.

| **Reagent** | **Vendor** | **Catalog Number** | **RRID** |
| --- | --- | --- | --- |
| IFNγ ELISPOT set | BD Biosciences | 551083 | AB_2868922 |
| Fix/perm kit | BD Biosciences | 51-2090KZ | AB_2869008 |
| FC Block | BD Biosciences | 553142 | AB_394656 |
| Mouse anti-IFNγ | BD Biosciences | 562020 | AB_395376 |
| Anti-CD45.2 | Biolegend | 109813 | AB_389210 |
| Anti-CD44 | Biolegend | 103031 | AB_2076206 |
| Anti-CD8 | Biolegend | 100803 | AB_312764 |
| Anti-NKp46 | Biolegend | 137607 | AB_10612749 |
| Anti-CD4 | Biolegend | 100527 | AB_312728 |
| Anti-Granzyme B | Biolegend | 515407 | AB_2562195 |
| Anti-F4/80 | Biolegend | 123123 | AB_893475 |
| Anti-PD1 | Biolegend | 109101 | AB_313418 |
| Anti-CD25 | Biolegend | 302611 | AB_314281 |

RRID: Research Resource Identifier
