# Supplementary material for: Foxp3 inhibitory peptide encapsulated in a novel CD25-targeted nanoliposome promotes efficient tumor regression in mice
Source: Acta Pharmacol Sin. 2024 Jul 29;46(1):171–83. doi: 10.1038/s41401-024-01338-0 (PMC11695603; doi:10.1038/s41401-024-01338-0)
Supplement: Supplementary file 9 — Supplementary Table S2 [file 41401_2024_1338_MOESM9_ESM.docx]

|  |  | **Control** | **P60**  **Low dose** | **P60**  **High dose** | **L-P60_750_** | **IL-P60_750_** |
| --- | --- | --- | --- | --- | --- | --- |
| **General Parameters** | **Weight**  **(g)** | 20.4 ± 0.6 | 20.7 ± 1.5 | 19.7 ± 0.2 | 20.8 ± 0.8 | 20.3 ± 0.3 |
|  | **Albumin**  **(g/dL)** | 4.2 ± 0.1 | 4.3 ± 0.1 | 4.2 ± 0.2 | 4.3 ± 0.3 | 4.3 ± 0.2 |
|  | **Glucose (mg/dL)** | 157.3 ± 19.4 | 187.2 ± 15.8 | 167.8 ± 2.0 | 181.8 ± 16.4 | 178.9 ± 4.4 |
|  | **Triglicerids (mg/dL)** | 141.3 ± 3.4 | 167.2 ± 15.6 | 109.4 ± 0.6 | 160.4 ± 35.5 | 142.5 ± 17.2 |
| **Hepatic Function** | **ALP**  **(U/L)** | 164.1 ± 54.5 | 152.0 ± 3.2 | 152.7 ± 8.1 | 181.1 ± 26.8 | 198.0 ± 7.0 |
|  | **ALT**  **(U/L)** | 22.4 ± 3.9 | 23.3 ± 4.4 | 27.3 ± 5.0 | 22.8 ± 0.1 | 28.2 ± 3.2 |
|  | **AST**  **(U/L)** | 59.6 ± 12.1 | 56.7 ± 7.8 | 98.5 ± 33.4 | 56.8 ± 5.9 | 78.4 ± 23.5 |
| **Renal Function** | **Urea**  **(mg/dL)** | 45.2 ± 9.0 | 45.9 ± 1.5 | 44.2 ± 12.6 | 41.6 ± 2.0 | 38.7 ± 7.8 |
|  | **Creatinin (mg/dL)** | 0.19 ± 0.05 | 0.17 ± 0.06 | 0.23 ± 0.09 | 0.18 ± 0.17 | 0.28 ± 0.19 |

**Table S2.** Parameters associated with physiological functions and body weight measured at the end of the different treatments. Data represent the average ± standard deviation.
